# A New Landscape of Human Dental Aging: Causes, Consequences, and Intervention Avenues

**DOI:** 10.14336/AD.2022.1224

**Published:** 2023-08-01

**Authors:** Yajia Xie, Shuang Chen, Lu Sheng, Yu Sun, Shangfeng Liu

**Affiliations:** ^1^Shanghai Key Laboratory of Craniomaxillofacial Development and Diseases, Shanghai Stomatological Hospital, Fudan University, Shanghai, China.; ^2^Department of Endodontics, Shanghai Stomatological Hospital, Fudan University, Shanghai, China.; ^3^CAS Key Laboratory of Tissue Microenvironment and Tumor, Shanghai Institute of Nutrition and Health, Chinese Academy of Sciences, Shanghai, China.; ^4^Department of Pharmacology, Institute of Aging Medicine, Binzhou Medical University, Yantai, Shandong, China.; ^5^Department of Medicine and VAPSHCS, University of Washington, Seattle, WA 98195, USA.

**Keywords:** dental aging, age-related pathology, cellular senescence, anatomical and cytological changes, SASP, chronological aging

## Abstract

Aging is accompanied by physical dysfunction and physiologic degeneration that occurs over an individual’s lifetime. Human teeth, like many other organs, inevitably undergo chronological aging and age-related changes throughout the lifespan, resulting in a substantial need for preventive, restorative as well as periodontal dental care. This is particularly the case for seniors at 65 years of age and those older but economically disadvantaged. Dental aging not only interferes with normal chewing and digestion, but also affects daily appearance and interpersonal communications. Further dental aging can incur the case of multiple disorders such as oral cancer, encephalitis, and other systemic diseases. In the next decades or even hundreds of years, the proportion of the elderly in the global population will continue to rise, a tendency that attracts increasing attention across multiple scientific and medical disciplines. Dental aging will bring a variety of problems to the elderly themselves and poses serious challenges to the medical profession and social system. A reduced, but functional dentition comprising 20 teeth in occlusion has been proposed as a measurement index of successful dental aging. Healthy dental aging is critical to healthy aging, from both medical and social perspectives. To date, biomedical research on the causes, processes and regulatory mechanisms of dental aging is still in its infancy. In this article, updated insights into typical manifestations, associated pathologies, preventive strategies and molecular changes of dental aging are provided, with future research directions largely projected.

## 1. Introduction

**Figure 1.**
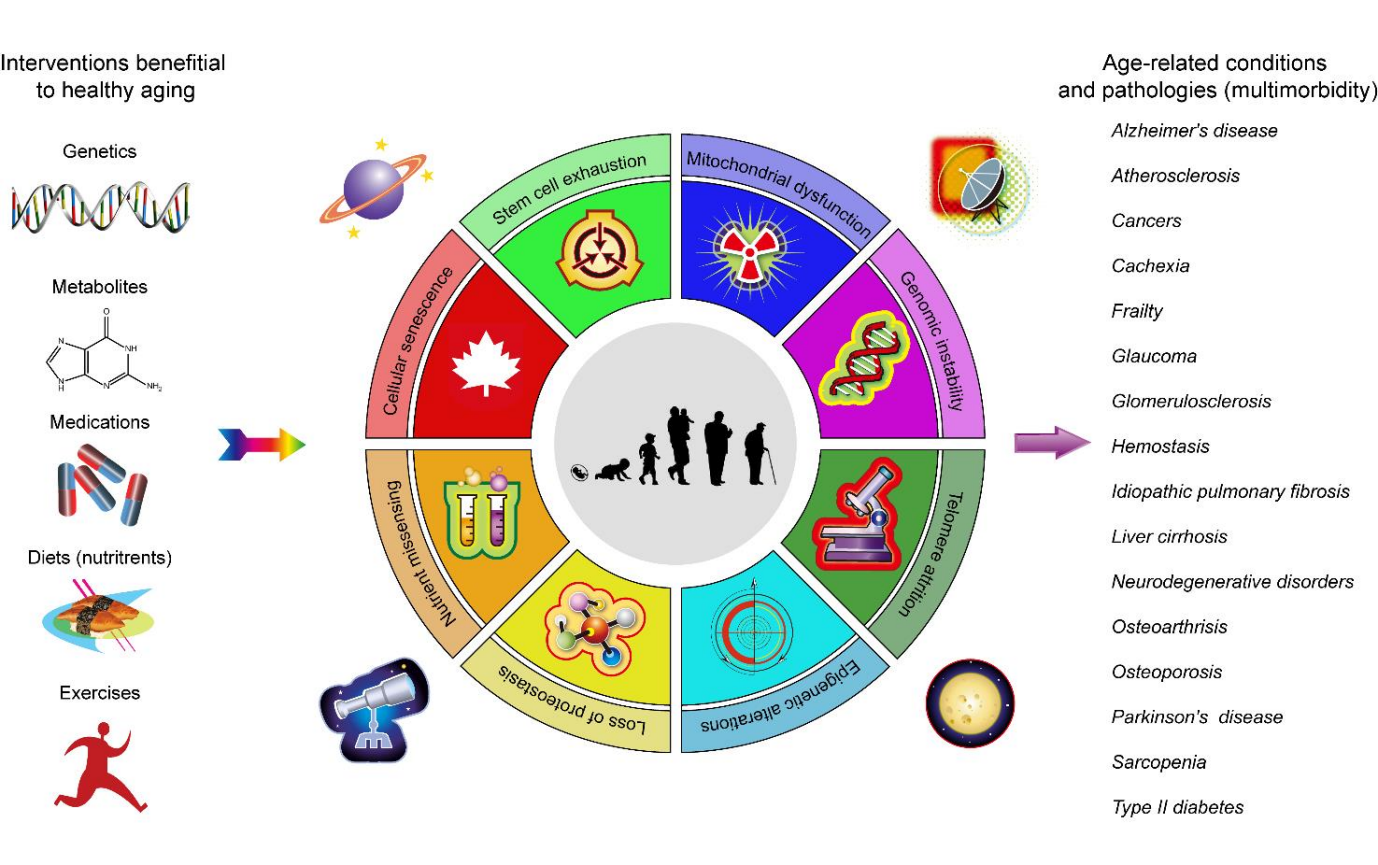
**Schematic representation of typical hallmarks of human aging, accompanying chronic diseases and potential interventions**. Aging is characterized by a set of typical hallmarks, including genomic instability, epigenetic alterations, mitochondrial dysfunction, loss of proteostasis, telomere attrition, deregulated nutrient sensing, stem cell exhaustion and cellular senescence. During chronological aging, the incidence of age-related pathologies augments in a progressive manner, most of which implicate cellular senescence. Treatment avenues involving genetic, dietary, or pharmacologic approaches represent promising intervention strategies to prevent or ameliorate aging symptoms. Part of the figure (mainly the circle) is adapted with permission from the reference [8]. Copyright year 2020, CellPress.

Aging is characterized by a progressive decline of the homeostasis and physiological function of multiple organs [1]. In the course of organismal aging, tissue degeneration and alternatively, inadequate tissue regeneration, are intimately linked to weakened immune responses and inefficient wound healing [2]. As a stable arrest of cell cycle progression, senescence was originally reported in human primary fibroblasts, which experienced consecutive passaging under culture conditions with a terminated proliferation [3]. Indeed, cellular senescence takes place comprehensively across almost all types of tissues *in vivo* [4, 5]. Although senescence represents a self-protective and active mechanism by preventing proliferation of aberrant or damaged cells after exposure to harmful stimuli, either inherent or exogenous, accumulation of senescent cells in aged tissues and organs can form a pathologically favorable niche, allowing organismal aging and the onset and development of various age-related diseases, a typical feature of organismal aging [6-8] (Fig. 1).

The activation of persistent or irreparable DNA damage response (DDR), telomere attrition, appearance of lipofuscin, and upregulation of p16 and p21 are considered as major features of senescent cells [9, 10]. Particularly, an enhanced activity of the lysosome enzyme, senescence-associated beta-galactosidase (SA-β-Gal), is widely accepted as a marker of increased lysosomal content during cellular senescence [11]. On the other hand, aging is related to the decrease of autophagy activity [12]. Autophagy represents a highly conserved mechanism to promote cellular homeostasis, which functionally removes defective, disabled, or redundant organelles and protein congeries. Decreased autophagy in older individuals is often associated with several age-related conditions, such as cardiovascular diseases, neurodegenerative disorders and metabolic symptoms [13].

Senescent cells frequently alter their surrounding microenvironments, mainly by producing and secreting a large number of pro-inflammatory molecules, a process widely known as senescence-associated secretory phenotype (SASP) and occurs in a non-cell-autonomous manner [14, 15]. The SASP is usually composed of multiple soluble factors, including but not limited to interleukins, chemokines, growth factors, metallo-proteases, and insoluble extracellular matrix components [16]. These molecules can markedly affect neighboring cells by activating transmembrane receptors and engaging their downstream signal transduction pathways [17].

In the course of aging the immune competency gradually declines, a process that is now termed as immunosenescence and causes increased susceptibility of older individuals to various microbial infections to tooth tissues [1]. Simultaneously, chronic pain, a condition defined as continued pain symptoms lasting more than 3 months regardless of the specific cause or site, has been reported among 38.5% of a cohort composed of 1141 older adults, and is more common among females and among adults over 85 years of age [18]. In dentistry, chronic pain in the elderly is largely attributed to several factors, such as neuropathy and immune system deficiency [19]. However, regenerative dentistry remains yet underdeveloped, although various dental pathologies and disorders affect virtually all the world’s population, particularly those aged [20] (www.euro.who.int/en/health-topics/disease-prevention/oral-health/data-and-statistics).

It has been well documented that dental pulp has a prominent regenerative capacity. In the case of tooth injury, odontoblasts, which are the pulp-derived cells responsible for dentine production, tend to degenerate and are subsequently replaced by undifferentiated mesenchymal cells that migrate to the affected site from the deeper pulp regions [21]. These cells differentiate into new odontoblast-like cells and produce reparative dentine [21, 22]. Dental pulp stem cells (DPSCs) exhibit high proliferative activity and can differentiate into either adipogenic, chondrogenic, myogenic, neurogenic, odontogenic, osteogenic, or vascular lineages [21, 23]. In this article, we aim to deliver an updated outline of dental aging-associated degenerative changes occurring in response to an array of pathophysiological events, and to analyze the relevant causes and major consequences. As experimental therapeutic approaches intimately related to age-dependent senescence constitute a novel and promising field of translational research, we chose to briefly propose future directions by summarizing the advantages and disadvantages of potential therapeutic approaches including those stem-cell-based for the global elderly.

## 2. Dental aging-related diseases

With the aging of the human body, various oral diseases began to arise gradually, in a manner similar to that of the vast majority of other organs. Many oral health problems are often overlooked, leading to delays of medication. During the aging process of various organs, immune function declines, resulting in a variety of oral pathologies, while systemic disorders such as hypertension and diabetes can co-exist and affect each other, gradually worsening the health status and life quality of the elderly.

The dental status of elderly patients is an important factor affecting the quality of their lives [24]. As one of the major organs of the human body, teeth also have their own age and aging process. Aging teeth are found to have the capacity to lead to a variety of diseases. Caries, periodontitis, incomplete fracture of teeth in the elderly and trigeminal neuralgia are common dental aging-related disorders (Table 1), among which caries (especially root caries) and periodontal disease are the main issues of the elderly. A systematic review showed that the average incidence of root caries in the elderly was about half of the total population [25]. According to a survey of hospitalized elderly people in Chennai, India, the prevalence of caries is 67.3%, and the prevalence of periodontal disease is 51.3% [26].

Consistent with the case of cancer, the incidence of which in the United States was 11 times higher for people older than 65 years of age than those younger than 65, it was estimated that among 42, 400 new cases of oral and pharynx cancer, nearly 50% of cases were in those aged 65 years and older [27]. As the population becomes older, the prevalence of head and neck cancer in people aged 65 and above in the Western world will increase by 27% from 2020-2030 [28]. It was shown by the statistics of inpatients in oral and maxillofacial surgery that the leading disease over 65 years old was oral and maxillofacial tumors, followed by salivary gland diseases, and malignant tumors were significantly more than benign tumors [29]. Oral and maxillofacial malignant tumors mainly occur in the mouth, followed by salivary glands and neck, the main tumor sites of oral cancer were tongue and palate, accounting for about 40.2% [30]. The pathological type is mainly squamous cell carcinoma, and its incidence is 53.5%-75.8% [31]. The most frequent primary origins for oral cancer metastasis do not correspond to the relative frequency of the primary tumors in the elderly population, suggesting that metastatic spread is not random. Despite the involvement of gingiva and jawbones in the majority of metastasis events, some other oral mucosal locations may be involved. Among elderly patients with oral and maxillofacial tumors, hemangiomas accounted for the highest proportion (28.05%), followed by papillomas (12.28%), for which the moon tongue and lips, respectively, are prone to [32].

**Figure 2.**
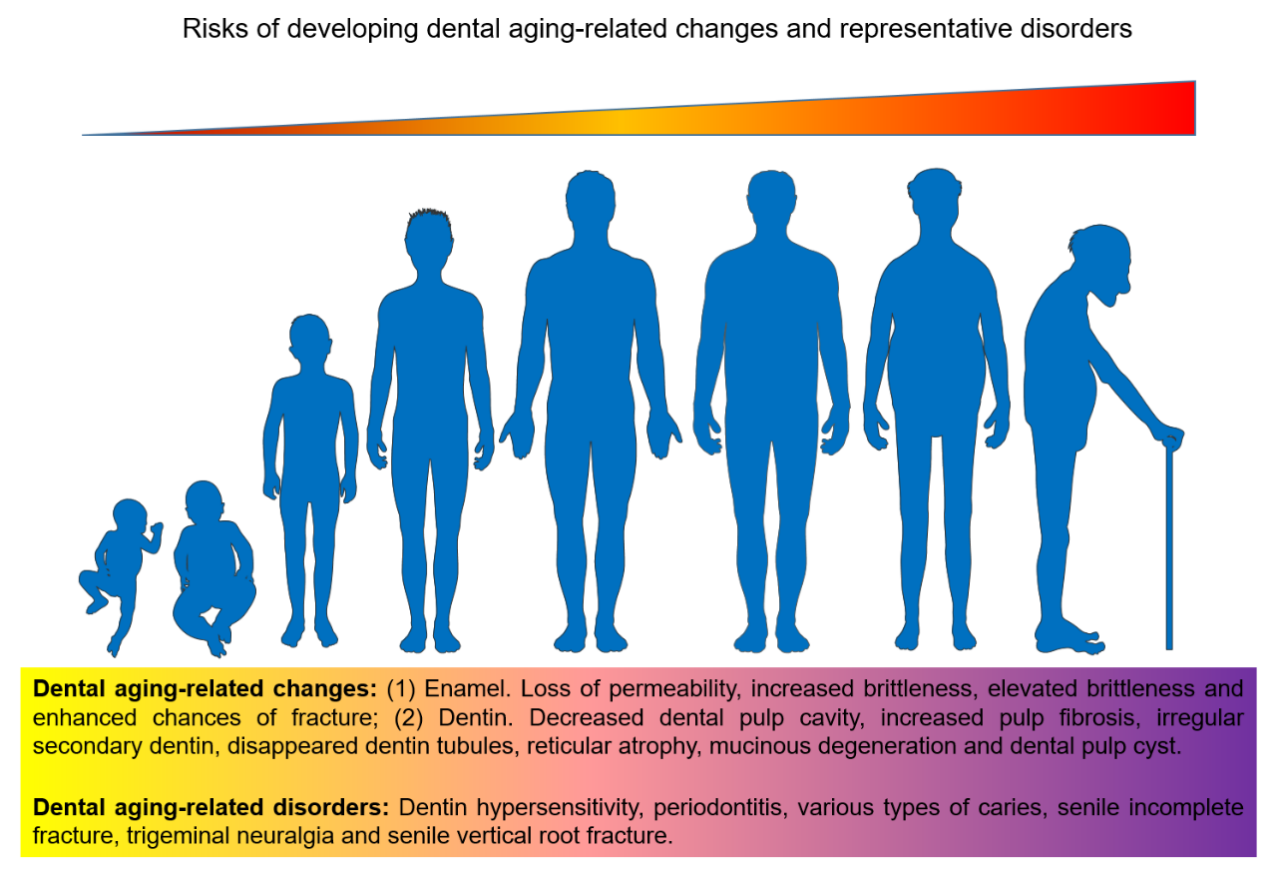
**Schematic illustration of pathophysiological risks of an individual in developing dental aging-related disorders**. In the time course of chronological aging, teeth are subject to a series of anatomical and/or cytological changes, factors that together contribute to human dental aging and related diseases. Dental aging-related changes typically observed in enamel and dentin, as well as representative dental aging-related disorders are exemplified.

As research continuously progresses, oral microbiota is found to play a key role in age-related systemic disorders (Table 2). Disorders of oral microbiome can cause pathologies of cardiovascular system, respiratory system, nervous system, endocrine system and reproductive system, respectively [33-38]. In the time course, generally, risks of developing dental aging-related diseases continue to increase until the end of an individual’s lifespan (Fig. 2). For example, a study uncovered 23 oral commensal bacteria within atherosclerotic plaques in patients that experienced carotid endarterectomy, catheter-based atherectomy or similar procedures, while 5 of them, namely *Campylobacter rectus*, *Porphyromonas endodontalis*, *Porphyromonas gingivalis*, *Prevotella intermedia* and *Prevotella nigrescens* are unique to coronary plaques [39]. Gingival tissues have multiple microbiota, which often causes inflammatory reactions and plays a critical role in assessing the susceptibility of patients to periodontal disorders [40]. The correlation between chronic periodontitis and the risk of malignancy *via* inflammation of the affected epithelium has been studied widely, with chronic periodontitis exerting systemic influence on development of multiple cancer types that involve consumption of alcohol and smoking, as well as diet, age and gender [40]. Specifically, risk factors for oral squamous cell carcinoma (OSCC) apart from alcohol and tobacco use, encompass poor oral hygiene, inadequately fitting denture-associated inflammation, insufficient nutrition and chronic infections caused by bacteria, fungi or viruses [41]. However, most cases of OSCC are detected at advanced stages, resulting in poor prognosis. A study focusing on microbiota differences between normal individuals, epithelial precursor lesion patients and cancer patients with distinct lifestyles including betel chewing and smoking, unmasked that the oral microbiome compositions of five genera, *Bacillus, Enterococcus, Parvimonas, Peptostreptococcus*, and *Slackia* of these individuals are markedly different [42].

**Table 1:** Human dental aging-related diseases.

Diseases	Pathogenesis	Clinical manifestations	Clinical treatments
**Attrition**	There is a slow progressive loss of dental hard tissue during mastication. Due to poor occlusal relationship, heavy tooth burden and other reasons, some elderly people are more prone to wear.	Enamel uniformity thinning, or pit-like performance, often towering cusps, sharp edges.	Eliminate pathogenic factors; desensitization treatment; occlusal reconstruction; repair missing teeth and restore occlusal balance.
**Dentin hypersensitivity**	Caused by gingival retraction, wear, tooth fracture, dental caries and so on.	Irritation pain, mechanical stimulation caused by the most significant symptoms, followed by chemical and temperature stimulation.	Etiological treatment, drug desensitization treatment, repair treatment, laser therapy, root canal therapy in severe cases [150-152].
**Periodontitis**	Periodontitis is an inflammatory reaction caused by plaque microorganisms and their products acting on periodontal tissue for a long time.	Gingival inflammation and enlargement; periodontal pocket formation; alveolar bone destruction and resorption; tooth loosening and displacement	Non operative periodontal treatment; periodontal tissue engineering [153-160]
**Quiescent caries**	The cariogenic environment disappeared, and the caries no longer progressed.	Dark brown, as hard as normal enamel, with a shiny surface	Quiescent caries need no treatment [161]
**Root caries**	Root exposure and plaque accumulation are caused by aging changes, which lead to demineralization, dissolution, and destruction of root hard tissue.	It often occurs on the cheek and tongue of the root of the tooth, showing a "shallow disc", sometimes forming circular damage around the whole root of the tooth; mostly without conscious symptoms	Non-traumatic dental caries filling; drug therapy; filling repair [162-165]
**Senile cracked tooth**	Due to the uneven wear and tear of the teeth for a long time, the traumatic bite force is formed, which can deepen and widen the enamel plate at the bottom of the tooth pit and fissure in the direction of dentin.	Prolonged masticatory discomfort or occlusal pain can be accompanied by tooth hypersensitivity and pulp congestion; it is common in the first molars.	The severity of apical clefts ranges from mild, requiring no treatment to severe root canal therapy or even removal is necessary [166].
**Senile vertical root fracture**	Longitudinal root fracture often occurs in middle-aged and elderly molars, in which the mesial root of the lower collar first molar is the most common. The causes are mainly related to traumatic bite force, root development defects and so on.	Long term bite discomfort. The crown is complete, the maxillofacial surface may be severely worn, or the tip may be high and steep, and different degrees of widening images of longitudinal root fissure can be seen on the X-ray.	In most cases, it is often necessary to cut roots or pull out the affected teeth for multiple teeth.
**Trigeminal neuralgia**	Trigeminal neuralgia is usually distributed along the cephalic nerve, and the cause of the pain is uncertain.	Intermittent severe pain in the distribution area of trigeminal nerve in unilateral or bilateral face	Drug treatment: carbamazepine, phenytoin sodium, etc.; surgical treatment [167-169].

Dysbiosis of the oral microbiome plays a critical role in the onset and development of periodontitis, displaying a shift to overgrowth of pathobionts in the normal microflora with elevated local inflammation [43]. As a common pathogen, *Fusobacterium nucleatum* (*F. nucleatum*) significantly overgrows in the case of periodontitis and has also been linked to various systemic disorders. Earlier studies reported the detection of antibodies against *F. nucleatum* in the circulating blood of patients with Alzheimer's disease (AD) or cognitive impairment (CI), but there is a lack of causal relationship and a plausible mechanism connecting these two conditions. A recent study revealed that *F. nucleatum* activates microglial cells, causing morphological changes, accelerated proliferation and enhanced TNF-α and IL-1β expression in microglial cells [43]. Importantly, *F. nucleatum*-caused periodontitis promotes exacerbation of the Alzheimer's symptoms in 5 X FAD mice, suggesting a likely link between a periodontal pathogen and the AD. Further, community individuals with AD often experience oral diseases and neuropsychiatric symptoms (NPS) in the course of disease progression. A study disclosed that decayed, missing and filled teeth, loss of attachment, plaque index, oral health behavior, pro-inflammatory cytokines, and salivary bacterial composition significantly differ among individuals diagnosed with AD, mild cognitive impairment (MCI) or subjective cognitive decline (SCD), three groups recruited in parallel [44]. The authors noticed that these parameters were generally poorer in the AD group rather than MCI and SCD groups and proposed targeted dental care and oral-related stress control as a valuable solution for NPS management in future clinics.

A number of epidemiological studies and intervention trials have attempted to demonstrate the relationships between maternal periodontal disorders and adverse pregnancy outcomes (APO), for which periodontal diseases are considered an important risk factor, including fetal growth restriction, gestational diabetes, low birthweight, preterm birth and pre-eclampsia [45]. For instance, a new study exploring the levels of periodontal red-complex bacteria (RCB) in pregnant pre-eclamptic women with periodontal diseases, potentiating the pathophysiology of a bacterial association in both periodontitis and pre-eclampsia [46]. However, restraining the periodontal bacterial load in pregnant women *via* good oral hygiene can significantly reduce the putative risk of adverse pregnancy outcomes including pre-eclampsia [46]. Thus, orisbacteria in saliva could be exploited as a non-invasive diagnostic indicator for early detection of oral and systemic disorders, while other therapeutic solutions including antimicrobial photodynamic therapy, antimicrobial peptides probiotics, green tea polyphenol and cold atmospheric plasma therapy are used to inhibit the growth of biofilm formation by oral bacteria [47].

**Table 2:** Oral microorganism-related systemic disorders.

Diseases	Pathogenesis
**Adverse pregnancy outcomes, APOS**	Adverse pregnancy outcomes including preterm birth (PTB), low birth weight (LBW) and pretermlow birth weight (PLBW) have been found to be correlated with changes in oral microflora [170]
**Alzheimer's disease, AD**	A large number of studies reported that people with periodontitis have an increased risk of developing AD [171], and those with AD or dementia have impaired oral health, as a result of cognitive decline, and are more prone to develop chronic oral diseases, such as periodontitis, tooth loss, and mucosal lesions [172-175].
**Atherosclerosis**	The pathological mechanism of atherosclerosis caused by oral microbiome is its colonization of atherosclerotic plaques and systemic inflammation caused by oral infection. The oral microbiome colonized on atherosclerotic plaque includes Streptococcus, Veronicus, Porphyromonas gingivalis, Actinobacillus actinomycetes, Treponema denticola, Fusobacterium nucleatum, Forsystenella and Neisseria [176].
**Breast cancer**	A meta -analysis of available epidemiological studies provided evidence of a modest 19% higher risk of developing breast cancer for individuals with periodontal disease compared to those without periodontal disease [177].
**Chronic obstructive pulmonary disease** **(COPD)**	Poor oral hygiene and periodontal conditions significantly increase the risk of COPD and are associated with frequent episodes of COPD. Of note, the dental plaque may lead to further aggravation of COPD [178].
**Colorectal cancer**	There are significant differences in the abundance of Clostridium nucleatum and Clostridium labile in oral cavity between normal people and patients with colorectal cancer. Periodontal pathogens such as Clostridium nucleatum may promote the progression of colorectal cancer through an inflammatory microenvironment [179].
**Diabetes mellitus**	The biodiversity and phylogenetic diversity of oral flora in diabetes and prediabetes were significantly lower than those in normal controls and related to the increase of pathogenicity of hyperglycemic flora. Periodontitis increases the risk of diabetes caused by infection or inflammation. To the contrary, diabetes also affects the progression of periodontitis [180].
**Esophageal carcinoma**	The detection rate of Porphyromonas gingivalis in the lesion area of esophageal squamous cell carcinoma was as high as 61%, while the detection rate in the adjacent area was only 12%, with the normal area not detected [181].
**Gastric cancer**	Poor oral health is associated with an increased risk of precancerous lesions of gastric cancer. The proportion of Neisseria and Haemophilus in the oral cavity of gastric cancer patients was significantly lower than that of healthy people [182].
**Human immunodeficiency virus**	HIV infection is associated with a series of oral diseases. Viremia in untreated patients is associated significantly with an increase in the proportion of potentially pathogenic viremia, Proteus, megaclobacter and campylobacter, as compared with healthy controls [183].
**Infective endocarditis**	Infective endocarditis is a serious infectious condition caused by bacteria and occurs on the inner membrane of the heart wall or the heart valve, often caused by bacteremia. More studies showed that periodontitis is a risk factor for infective endocarditis [184-186].
**Inflammatory Bowel disease**	Inflammatory bowel disease (IBD) is one of the earliest diseases related to oral microbiome. The intestinal vegetation of oral bacteria is considered to be widely involved in the occurrence and development of inflammatory diseases [187])
**Liver cancer**	Liver cancer is a common malignant tumor worldwide with high morbidity and mortality. Multiple tooth loss caused by periodontitis increases the risk of primary liver cancer [188].
**Liver cirrhosis**	Liver cirrhosis is caused by many chronic liver diseases. Compared the changes of intestinal flora in 98 patients with liver cirrhosis and 83 healthy individuals by macrogenomics, approximately 54% of the colonies with different abundance expression were oral microorganisms, indicating origination of part of the abnormal intestinal flora in patients with liver cirrhosis from the oral cavity [189].
**Lung cancer**	Among people without smoking habits, the incidence of lung cancer in patients with severe periodontitis has increased by 2.5 times. The saliva samples of patients with lung cancer were significantly higher than those of normal controls, suggesting that they may be biomarkers for early detection of lung cancer [190]
**Miocardial infarction**	Oral bacterial DNA could be detected in coronary artery thrombus aspiration in patients with myocardial infarction, in which the DNA of root canal infection typical bacteria (oral streptococcus) accounted for 78.2% of thrombus aspiration, with periodontal pathogens accounting for 34.7% [191].
**Obesity**	Obesity is associated with oral microbiome. The changes of salivary microbiota may be a systemic response caused by obesity, and the changes of some bacteria in saliva may be involved in the immune inflammatory process of obesity [192]. Thus, the species of oral bacteria may be used as a biological index of overweight.
**Obstructive sleep apnea hypopnea syndrome, (OSAHS)**	Changes in the gut microbiota have a pathophysiological role in OSAHS and may be associated with the pathophysiology of metabolic comorbidities in patients diagnosed with OSAHS [193].
**Pancreatic cancer**	Gastrointestinal cancer is closely related to oral microbiome. Periodontal history and the presence of circulating antibodies against some oral pathogens are linked to the occurrence of pancreatic cancer. Porphyromonas gingivalis and Actinomyces could increase the risk of pancreatic cancer in a large-scale case-control study [194]. The risk of pancreatic cancer in patients with seropositive Helicobacter pylori would increase by 38% (OR=1.38, 95% CI: 1.08~1.75), which suggests that Helicobacter pylori may play a role in the occurrence and development of pancreatic cancer [195].
**Poly cystic ovary syndrome, (PCOS)**	PCOS is a common female endocrine disease with unknown etiology. The composition of microbial components in PCOS saliva was reported, and the number of actinomycetes in the saliva of PCOS patients declined [196]. In addition, the difference was more significant in PCOS patients with gingivitis.
**Rheumatoid arthritis (RA)**	RA autoimmunity can be triggered or enhanced by specific oral bacteria that cause periodontal disease, in which Gram-negative bacteria Porphyromonas gingivalis may play important roles [197, 198]. In addition, increased prevalence of periodontitis is observed in patients with RA [199].

## 3. Dental aging-related anatomical changes

**Figure 3.**
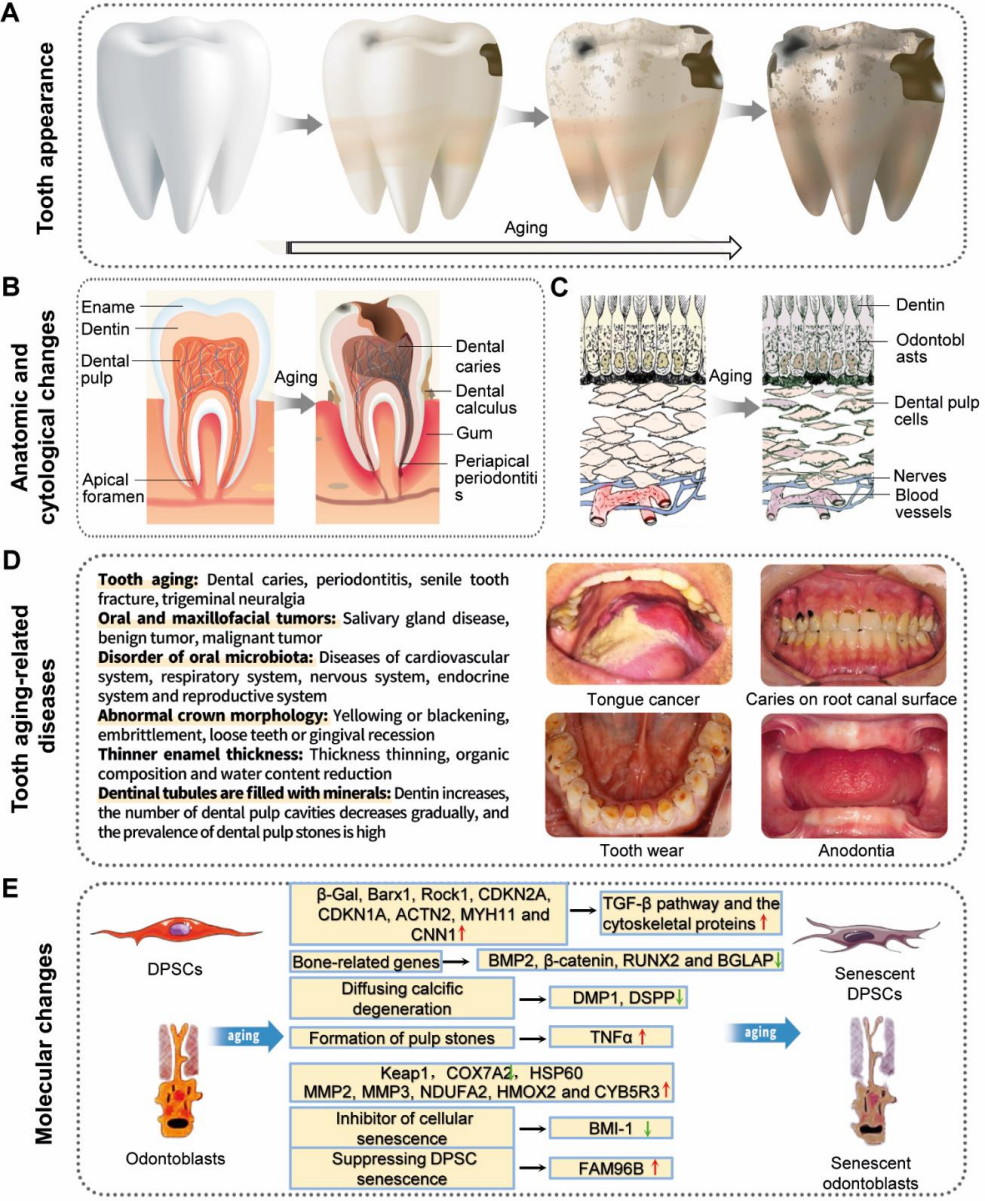
**Graphic profiling of human tooth aging and related pathophysiological changes**. (**A**) appearance of aging teeth. (**B**) anatomic changes in aging teeth. (**C**) histological changes in aged tooth tissues; (D) major diseases associated with human dental aging and associated phenotypic alterations; E, molecular biological changes in human dental aging.

The anatomical changes resulting from aging teeth in humans (Table 3) are basically similar to those of the skeletal system (Figure 3A, 3B). Aging teeth can cause symptoms such as yellow or black teeth, brittle, sensitive, loose teeth, and receding gums. The hard tissues of tooth body include dentin, enamel, and cementum, among which dentin and enamel are the main components that bear chewing pressure. Enamel is the hardest and toughest mineralized tooth tissue, maintaining its functions throughout a lifetime, despite a few millimeters thick with minimal regenerative capacity [48]. With the increase of age, the thickness of enamel becomes thinner, the organic composition and water content further decrease, the weak parts such as pericardium become calcified, and the structure becomes denser [49, 50]. Furthermore, the permeability of enamel is lost, the brittleness is increased [51], while the levels of nitrogen content and neck fluoride are elevated [52, 53]. Enamel hardness showed a decreasing trend from the enamel surface to the dentin-enamel junction (DEJ) [54, 55], and the teeth of elderly patients (55 years or older) had higher elastic modulus and surface hardness than those of younger patients (18 to 30 years old) [56]. Although enamel properties can vary with tooth origin, tooth type and tooth surfaces, different teeth and enamel depths exhibit differential susceptibility to age-related erosions, making it necessary to standardize enamel-related experiments involving the measurement by surface microhardness loss and calcium release [54]. A recent study applying unsupervised machine learning tools to pursue further understanding of relationships between the composition and mechanical behavior of aging enamel suggest that unsupervised learning approaches can reveal complicated structure-property relationships of dental tissues and help gain insights into the materials science of aging and its consequences [57].

**Table 3:** Dental aging-related phenotypic changes.

Different parts of teeth	Dental aging phenotypes
**Enamel**	Loss of permeability, increase of brittleness and the obvious calcification of organic matter between enamel prisms, can result in brittleness and fracture; elevation of nitrogen content in enamel leads to the blackening of teeth; there are changes in enamel structure and refraction, which may be related to changes in enamel structure; aspartic acid in enamel shows more and more racemization, which may become an aging biomarker; fluoride levels in enamel are increased; however, wear rate of enamel is not an accurate age measurement method.
**Cementum**	The width of cementum increases, mainly at the top. In advanced stages, the deposition of dentin and cementum may be increasingly complete. The rhythmic accumulation of cementum (annular) has been used to determine the age of animals.
**Dental arch width**	In the process of aging, the mandibular molars tend to move closer to the middle, the first premolars and canines tend to move lingually, and the dental arch narrows left and right.
**Dental pulp and cytological changes**	Dental pulp fibrosis, reticular atrophy, mucinous degeneration, dental pulp cyst; fat droplet deposition and replacement; calcification of medullary cavity and root canal; hyaline degeneration is usually the intermediate stage of pulp calcification; the circulation and innervation is damaged, and the number of nerve axons in dental pulp decreases; reduction of capillaries and lymphatic vessels; elderly dental pulp stem cells have few cytoplasmic fibers and organelles; the remaining odontoblasts become smaller, flattened and shrunk; mitochondrial molecules gather around a large amount of autofluorescence lipofuscin deposition.
**Dentin**	The pulp cavdentinscomes smaller due to the continuous deposition of secondary dentin; The secondary dentin becomes irregular, the dentin tubules gradually disappear, and the formation of transparent dentin begins at the root tip. With increased age, the dentin gradually develops to the crown.
**Periodontal tissue**	Periodontal tissue atrophy is the main feature of aging-related dental changes. It can make the whole tooth have extensive gingival margin and alveolar bone, shrinked back at the same time, and exposed root. Under normal circumstances, this physiological change is called senile periodontal atrophy, which can shrink by about 1 mm at 60-70 years of age. Severe periodontal atrophy is mainly caused by secondary pathological factors. The thickness of periodontal ligament in normal young individuals is 0.21 mm, which can be reduced by 1/3 after the age of 60, a change that is mainly related with the reduction of masticatory function.

A "dissolution and precipitation" mechanism were proposed, which may interpret tubule occlusion and sclerotic dentin formation based on the chemical composition and crystal size changes of the minerals deposited in the lumen [58, 59]. With age, once the lumen is filled with minerals, transparency will appear, which is called transparent dentin or hardened dentin. Transparent dentin starts from the top of the root and usually extends to the crown dentin [58], which is different from the pathological transparency of caries and belongs to natural aging, with its flexibility, viscoelasticity, bending strength and cyclic mechanical properties significantly declined [58, 60]. Compared with young dentin, the stress generated by fatigue cracks in old dentin is lower, while the growth rate becomes significantly higher [60, 61]. In the young dentin, elastic resistance of the tissue can prevent the crack from extending to the long axis of the dentin tubule and the vertical direction of the pulp cavity [62]. In the aged dentin, however, due to the decrease of elastic limit, the cracks are more likely to extend along the tooth long axis and dentin tubule long axis [63]. Therefore, aging leads to an increase in the elastic modulus and hardness of teeth, a greater change in the mechanical properties of the inner dentin, a decrease in fracture toughness and strength, an increase in brittleness, a decrease in the ability to resist cracks under stress and elevated susceptibility to fatigue fracture [64]. Despite significant drawbacks includ tooth discoloration, intracanal use of the triple antibiotic paste (TAP) may provide advantages. A recent study demonstrated the association of this practice with scaffolds containing minocycline or doxycycline, and the production of color changes similar to those of dentin when compared with their respective TAP systems, although doxycycline-related discoloration was less dramatic [65].

As a biomarker of aging, aspartic acid in enamel shows more and more racemic activity [66], resulting in irregular continuous deposition of dentin, hardening of dentin tubules and gradually smaller pulp cavity [67-71]. Some studies have reported a high prevalence of dental pulp stone (DPC) in the elderly [72, 73]. However, the association between DPC and age remains controversial [74-76]. Odontoblasts undergo degenerative changes, atrophy and disappearance in most areas of the dental pulp [77]. Of note, dentin features and mechanical properties tend to change with age, with alterations occurring spatially within teeth depending on tubule orientation. Age-related mineral deposition within tubules and collagen maturation in intertubular dentin can markedly compound the spatial effects on mechanical properties of dentin [78]. The dentin tubules are enriched with minerals, the lumen disappear gradually (‘occlusion’) and the dentin around the tubules increases with age. The formation of transparent dentin (‘sclerosis’) begins from the root tip and gradually develops towards the crown [77, 79]. In the course of aging, dentin tends to lack facility to remodel or repair, except at the pulp periphery, where odontoblasts secrete matrix, predentin, especially at the mineralization growth front [80]. More importantly, the post-mitotic odontoblasts succumb to age-related autophagic activity to decline in function, while they continue to secrete dentin matrix until injury or death [80, 81].

Affected by genetic, epigenetic, and environmental factors, the hard tissue of teeth will be abnormally developed or mineralized. Aberrant enamel structure, such as dental fluorosis, usually involves permanent tooth changes, ranging from white spots on light enamel to heavy loss of normal tooth morphology (Fig. 3A). The ultrastructure showed poor enamel mineralization, but the enamel surface was over mineralized, the direction of enamel column was irregular, and the arc structure of enamel dentin boundary was more obvious [82]. Atomic force microscopy showed that there was a positive correlation between the degree, roughness and absolute depth of fluoride damage to dental enamel crystals, and it was also related to histological grading and clinical manifestations of dental fluorosis according to the Thylstrup-Fejerskov classification [83]. Dental fluorosis has clinical and histological manifestations similar to those of other types of enamel mineralization and hypoplasia. The United States Public Health Service recommends a fluoride concentration of 0.7 mg/L of water to prevent caries, one of the most prevalent chronic diseases, while reducing the risk of dental fluorosis, which mainly correlates with systemic consumption of fluorides especially in the early life [84].

Human dentin can be considered as a hierarchical material with multilevel micro-/nano-structures, comprising tubule, perti-tubular dentin (PTD) and intertubular dentin (ITD) as the principal constituents at microscale, while the PTD and ITD are further composed of collagen and hydroxyapatite crystals with different volume fractions at nanoscale [85]. Abnormal dentin structure, such as hereditary opalescent dentin, usually has color changes such as blue, yellow and amber light transmittance of teeth [86]. The ultrastructure showed that the dentin structure of a thin layer of near enamel was largely normal with aging, while other dentin structures markedly changed. For example, the number of dentin tubules was abnormal, the direction was disordered, the shape of tubules was irregular, the diameter of tubules became larger, and there were dentin areas without tubules [87, 88]. Although some studies show roughly normal scallop-like structure [89], other investigations have found that the enamel dentin boundary is linear, loses scallop-like structure, and even has large cracks between enamel and dentin [87].

Current research generally lags for anatomical changes of aging teeth, with related aging mechanisms largely unclear yet. Prior to interventions to teeth themselves to delay the aging process of teeth, only treatment with simple symptomatic alterations can be practiced. For caries, traditional filling and repair methods not only fail to completely restore the original function of the defective teeth, but cause secondary caries and micro-leakage, which is currently an unsolvable problem. Most dental tissue has little or no capacity of self-regeneration [90, 91]. With age, there is less organic substrate or water, and enamel displays a reduced capacity of regenerating itself after significant mineral loss [92]. Therefore, it is technically urgent to develop effective strategies to repair the teeth upon aging-related loss of dental functions. In recent years, tooth regeneration has attracted extensive attention based on the understanding of tooth growth mechanisms, natural tooth morphology regulation in human body and critical signaling pathways associated with tooth regeneration. Despite advances in dental tissue repair and techniques replacing missing teeth with prostheses, clinical treatments cannot regenerate tissues with natural teeth properties. In contrast, tooth tissue engineering (TTE), a promising method for dental tissue regeneration, holds the potential to form durable biological substitutes for soft and mineralized dental tissues [93]. To date, however, partial or complete tooth reconstruction remains a major challenge for future studies of dentistry and oral medicine [17-19].

## 4. Dental aging-related cytological changes

Dental pulp is a soft connective tissue, comprising fibroblasts, mesenchymal stem cells (MSCs), nerve fibers, odontoblasts and vessels, while this tissue is originally derived from dental papilla, an ectomesenchymal cell condensation in the developing tooth [94]. The dental papilla cells underlying the enamel epithelium can differentiate into odontoblasts, a process controlled by epithelial-mesenchymal interactions [95]. The cell density of dental pulp in 70-year-old individuals is largely half that of 20-year-old individuals, indicating a reduction in pulp restoration capacity [96]. Specifically, there is a decrease of 15.6% in crown odontoblast cells and 40.6% in root odontoblast cells, with reduced secretion activity and vacuolation of cells, further supporting that the repair ability of dental pulp tends to decline with age [52, 97] (Fig. 3C). In addition, dystrophic calcification in the central pulp of the coronal region and root canal is evident in older individuals, which might be due to their reduced pulpal blood flow [52, 98].

The volume of dental pulp decreases with age, mainly due to secondary dentin deposition throughout lifespan [99]. One of the most obvious characteristics of pulp aging is indeed the continuous secretion of dentin matrix by odontoblast cells, resulting in the reduction of pulp chamber [100]. With age, there are difficulties in endodontic treatment, because of the constriction of pulp chamber space by hyperplasia of secondary and tertiary dentin, as well as pulp stones and diffuse calcification in the radicular pulp [101]. In aged individuals, the pulp appears to be fibrosis-like, the number of pulp cells decreases, the pulp shrinks and becomes smaller and flatter [52, 66, 77, 102]. Fat deposition and substitution occurs [103, 104], arteriosclerosis changes take place in blood vessels, lymphatic vessels and nerves decrease, with supply impaired and mineralization increased [102, 105, 106]. Golgi complexes are shrinked in senescent pulp fibroblasts, while mitochondrial molecules cluster around abundant deposits of spontaneous fluorescence lipofuscin [107].

DPSCs represent a vital subgroup of the adult stem cell family and are considered as a critical source for dental pulp tissue regeneration. Although considerable efforts have been made to identify the DPSC origin, so far, the real origin remains obscure. It was proposed that DPSCs originate from pericytes, cells that reside in the perivascular niche of postnatal dental pulp [108, 109]. Over the past several years studies have indicated that neural crest cell (NCC)-derived MSCs contributing to tissue repair and regeneration are also derived from nerves [110]. Clonal DPSCs from neonatal murine tooth pulp can show multi-differentiation in neural crest lineage for adipocytes, chondrocytes, odontoblasts, neurons and smooth muscles, supporting the existence of neural crest-derived DPSCs with differentiation capacity into cranial mesenchymal tissues and other neural crest-derived tissues [111]. Another study indicated a substantial population of MSCs in the course of dental development, while the self-renewal and repair capacity of a tooth are conferred by peripheral nerve-associated glia. The authors demonstrated that glial cells generate multipotent MSCs producing pulp cells and odontoblasts, providing advanced insights into the dynamics of tooth organogenesis and growth [112].

Compared with other stem cells, DPSCs also have unparalleled advantages, including less ethical problems, simple acquisition, strong activity, aging slowly, low risk of tumorigenesis, small trauma and life-long access for most people. Moreover, origin and neural crest cells have brought therapeutic hope to patients with highly disabling conditions such as spinal cord injury, stroke or neurodegenerative diseases [113], and have become seed cells with considerable potential in tissue engineering. However, in the current actual clinical treatments, such as in the case of repair of an injured tooth, their therapeutic efficacy remains low, particularly for the elderly.

At present, functional pulp regeneration is one of the research hotspots, which requires not only the ability to form dentin (dentinogenesis) but also the capacity to restore dental pulp vasculature (blood vessels) and nerves to ensure proper cell nutrition and maintain viability, as well as to restore and sustain proper defence against potential pathogens encountered in pathophysiologic states such as pulpitis, periodontal disease, and trauma [114] (Fig. 3D). Vital pulp therapies to repair partially damaged dental pulp have been applied with some success in dental clinics [115]. So far, the gold standard material for vital pulp capping is mineral trioxide aggregate (MTA) [116]. More recently, Biodentine has shown great promise for applications in pulp capping [117]. In the past, regenerative endodontics therapies (RETs) have focused on partial regeneration of the dentin-pulp complex, with the goal of protecting the continuous growth of immature tooth roots, and to reduce the risk of fracture associated with traditional apexification procedures in thin and weak roots [118]. More recently, RETs have focused on whole pulp regeneration in an attempt to replace currently used synthetic material-based endodontic treatments that devitalize the tooth by filling the pulp chamber with gutta-percha (GP) or another synthetic material that blocks vital tissue formation [119]. In contrast to partial vital pulp therapy, whole pulp regeneration is focused on total pulp regeneration and revascularization of fully developed teeth by facilitating DPSC-derived odontoblast differentiation and pulp regeneration [120]. DSPC has been successfully isolated from inflamed dental pulp tissue, supporting the feasibility of whole pulp regeneration using autologous cell sources from infected dental pulp [121].

At present, the treatment methods used to repair tooth injury and loss largely depend on the application of synthetic materials to solve structural deficiency, without promoting biological activities such as blood transportation and nerve supply.

## 5. The landscape of molecular and cellular changes associated with dental aging

Senescent cell accumulation in aging tissues allows formation of favorable microenvironments for both the onset and progression of age-related pathologies. The response to gradual loss of genomic, epigenomic, proteomic and metabolic integrity in aging tissues can be triggered and controlled by two major tumor suppressor pathways: the p53-p21(CDKN1A)-retinoblastoma (RB) axis and the p16(CDKN2A)-RB branch [16]. Although these two pathways can independently arrest cell cycle progression, they can also mutually crosstalk in certain situations, particularly upon cellular senescence [122]. Although being arrested in an essentially irreversible status, senescent cells hold the potential to markedly change their surrounding microenvironments, as they secrete pro-inflammatory and matrix-degrading molecules, a process now known as the SASP [14]. In the local microenvironment, a full spectrum SASP expresses various soluble factors, including interleukins, chemokines, growth factors, metalloproteases, and insoluble extracellular matrix components, which together affect neighboring cells by activation of cell-surface receptors to engage intracellular signal pathways [8]. Recent studies suggest that these cell-non-autonomous processes are associated with many age-related oral conditions, including caries and periodontitis [1, 123].

DPSCs are considered as a critical source for dental pulp tissue regeneration, such as in the case of repairing an injured tooth, but their therapeutic efficacy remains limited, particularly in the aged individuals. A recent study investigated the differences in DPSC protein expression profiles using proteomics and bioinformatics approaches and identified 75 upregulated proteins and 69 downregulated proteins in DPSCs from elderly donors [124]. Specifically, high mobility group N1 (HMGN1), HMGN2, UCHL1 and the family with sequence similarity 96 member B homeobox gene (FAM96B) were associated with DPSC senescence, while FAM96B plays an important role in suppressing DPSC senescence and promoting osteogenic differentiation and proliferation [124]. Through RNA-seq and qPCR approaches, another study identified the homeobox protein Barx1 as a novel marker for DPSCs [125]. Further analyses with high throughput transcriptomic and proteomic analysis uncovered a set of markers for DPSC populations with accelerated replicative senescence, including CDKN2A, CDKN1A, ACTN2, MYH11 and CNN1 [125] (Fig. 3E). Specifically, expression of the transforming growth factor beta (TGF-β) pathway and the cytoskeletal proteins are increased in rapidly senescent DPSCs, suggesting a decline of stem cell properties and onset of terminal differentiation. Using metabolic flux strategy, the researchers further established a metabolic signature for these senescent DPSCs, which do show up before appearance of typical senescence-associated phenotypes [125].

The process of aging can also affect the ability of DPSCs to promote mineralization. A decreased osteogenic potential was recently observed in senescent human DPSCs, with the decline of differentiation potential towards mineralized tissues including bone and dentine associated with changes in expression of bone-related genes, such as BMP2, β-catenin, RUNX2 and BGLAP [126, 127]. Furthermore, the expression of DMP1 and DSPP, key odontogenic differentiation markers, is markedly decreased in DPSCs cultured in odontogenic medium derived from aged patients [127]. DMP1, a protein that regulates mineralization, translocates from nucleus to cytoplasm during cell differentiation, is eventually secreted to the extracellular space, where it regulates the nucleation of hydroxyapatite [128, 129]. Senescent DPSCs have relatively lower amounts of cytoplasmic DMP1 as compared to their proliferating counterparts during odontogenic differentiation, a tendency that indeed correlates with the diffusing calcific degeneration of the pulp and occurs as a response to aging [127, 130]. However, age-correlated features of DPSCs may be reversed upon exposure to appropriate stimuli and extracellular substrates. For example, DPSCs from aged individuals exhibit regenerative properties similar to those of DPSCs isolated from young patients, when cultured on nanostructured hydroxyapatite scaffolds and used *in vivo* to eliminate calvaria defects in rats [131].

**Figure 4.**
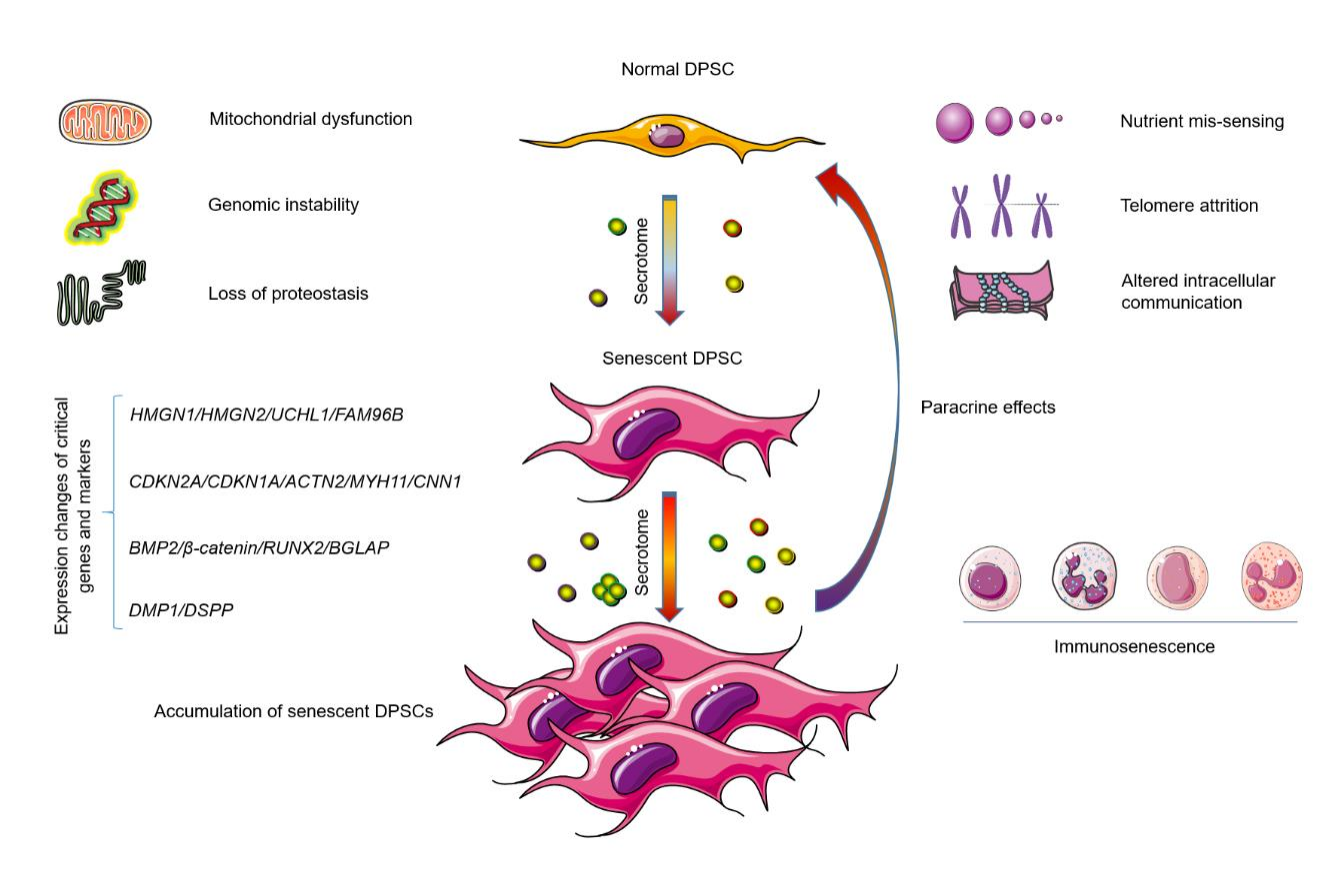
**Development of DPSC senescence, accumulation of senescent DPSCs in human tooth tissues and their pathological influences**. As a critical source for the regeneration of dental pulp tissues, DPSCs exhibit high proliferative activity and can differentiate into adipogenic, chondrogenic, myogenic, neurogenic, odontogenic, osteogenic, or vascular lineages, thus involved in a wide variety of essential activities to maintain tooth homeostasis. However, senescent DPSCs can generate diverse adverse effects that compromise tissue integrity and accelerate pathological progression. DPSC, dental pulp stem cells.

In the course of aging, secretion of a handful of senescence-associated factors, including matrix metalloproteinases (MMPs) may be changed [17]. The concentration of specific MMPs can increase significantly in inflamed pulp, particularly when compared with the normal pulp [132]. In pulp tissues from patients suffering from acute pulpitis, protein expression of MMP2 and MMP3 was considerably higher than those in the pulp from healthy donors, implying a possible role of MMPs in the case of pulp inflammation and/or tissue damage. Specifically, MMP3 may activate the expression of other MMP molecules, such as MMP1, 7 and 9, which are able to trigger the collagen degradation and eventually cause changes in the extracellular matrix, events that have been observed in tooth tissue pathologies including acute and chronic pulpitis as well as periapical lesions [133, 134]. The development of dental caries into the dental tissues is able to promote inflammatory cell accumulation within dental pulp. These cells secrete inflammatory cytokines such as tumor necrosis factor alpha (TNF-α), a molecule that enhances mineralization [135, 136], partially explaining the emergence of nucleation points, which potently drive the formation of pulp stones in teeth of the aged [137]. Together, these findings indicate that the aging process can substantially affect the DPSC secretome (Table 4) (Fig. 4).

## 6. Prevention of human dental aging

In the past 70 years, our global population and its age structure have been changing rapidly, a problem that is increasingly prominent and will continue to exist for the rest of the 21st century. The World Health Organization (WHO) estimates that the global population aged 65 or over is growing at a rate of 2.5%, and the number will reach approximately 1.5 billion by 2050 (www.nia.nih.gov/research/dbsr/global-aging) and 2.37 billion by 2100 [138]. This will clearly pose a major challenge to the healthcare system to ensure a healthy and long life in an aging society.

**Table 4:** Dental aging-related genes and their changes.

Genes	Functions in aged dental pulp stem cells (DPSCs)
**BMI-1**	An already known inhibitor of cellular senescence. The expression of BMI-1 (B lymphoma Mo-MLV insertion region 1 homolog) is decreased in senescent DPSCs [200].
**BMP2, BGLAP, β-catenin, RUNX2**	The osteogenic potential was decreased in senescent human DPSCs, with the decline of differentiation potential towards mineralized tissues including bone and dentine. There are aging-associated changes in expression of bone-related genes [127, 201].
**COX7A2, CyB5R3, HMOX2, Keap1, NDUFA2,**	The downregulated oxidation promotion-related proteins, including Keap1, COX7A2 and their regulated antioxidative molecules, NDUFA2, HMOX2 and CYB5R3 in aging DPSCs [202-206].
**DMP1, DSPP**	Senescent DPSCs have relatively lower amounts of cytoplasmic DMP1 as compared to their proliferating counterparts during odontogenic differentiation, a tendency that indeed correlates with the diffusing calcific degeneration of the pulp and occurs as a response to aging [127, 130].
**FAM96B**	FAM96B plays an important role in suppressing DPSC senescence and promoting osteogenic differentiation and proliferation [124].
**GTF2B, NFYB, NR3C**	As senescence-specific markers, β-galactosidase activity is increased in aging DPSCs, with the odontogenic differentiation potential disturbed [200, 207].
**NFYB, GTF2b, NR3C1**	NFYB, GTF2b and NR3C1 are the core transcription factors that affect the aging of DPSCs [126].
**HSP60, p16**	Expression of p16 is increased with age, whereas heat shock protein 60 (HSP60) is decreased in senescent DPSCs [208, 209].
**Rock1**	The abnormally high expression of Rock1 is related to the aging of HDPSCs [210].
**TGF-β**	Upregulated in rapidly senescent DPSCs, indicating a loss of stem cell properties and initiation of terminal differentiation [201].
**TNF-α**	TNF-α is a molecule that enhances mineralization [135, 211], partially explains the emergence of nucleation points, which potently drive the formation of pulp stones in aged teeth [207].

In 2016, the World Dental Federation adapted its definition of oral health from a narrow focus solely on disease, to a broader, multidimensional view that includes the ability to speak, smile, smell, taste, touch, chew, swallow, and convey a range of emotions through facial expressions with confidence and without pain or discomfort [139]. However, oral health used to be a neglected aspect of global health. With the inversion of population age structure and the expansion of the elderly relative to other age subgroups, the prevalence of oral diseases in the elderly will further increase. In addition, it is evident that the future dental healthcare system will be costly and largely insufficient to meet the mounting challenges.

Dental diseases and functional problems of the elderly mainly include dental caries, periodontal disease and edentulousness [140]. Untreated dental caries in permanent teeth affect approximately 2.5 billion of global populations [141] with the highest incidence at age 70 years. The prevalence of periodontal diseases is highest in those aged 60 years and higher, while advanced periodontitis, the sixth most common health concern worldwide, has profound implications on systemic health [142]. Worldwide, approximately 30% of adults aged 65-74 years are edentulous, whereby periodontal diseases are the primary cause, followed by caries, poor oral hygiene and multimorbidity, a condition commonly defined as the co-existence of multiple chronic disorders that are likely associated with severe tooth loss (Edentulism) by mis-nutrition or inflammatory activities [143, 144]. In addition, dental traumatic injuries caused by falls substantially affect older adults, and more than one in four older adults have a fall each year [145-147].

Most dental diseases are preventable. The most important prevention is to follow up every three months to evaluate emerging caries and soft tissue alterations. Fluoride varnishes need to be scheduled at least twice *per* year for patients with a high risk of caries and 1.1% sodium fluoride is recommended daily to further promote remineralization. The prevention of periodontitis is plaque control strictly, with chlorhexidine and lishdelin gargle widely accepted for periodontal care and plaque therapeutics of the elderly. In addition, a larger and easier to grasp toothbrush handle has been developed for the elderly with poor hand flexibility, which is convenient to guide brushing [148, 149]. We believe that these preventive measures will maximize the retention time of healthy teeth in the mouth and reduce the rate of edentulous patients. In recent years, with the continued advancement of new technologies, novel materials, and the proposal of the concept of minimally invasive dental treatment, dentists can carry out comprehensive treatment for common oral diseases of the elderly, promote oral and systemic health of the elderly and reduce the disease burden. Establishment and improvement of the prevention and therapeutic system, as well as treatment strategy of oral diseases in the elderly is one of the major goals for years to come.

At present, there is no specific strategy to reverse tooth aging, according to advances of scientific research. However, in daily life, the following methods may be followed to delay aging, prolong the service life of teeth as much as possible, and ensure the quality of lifespan and social dignity (in the order of significance in dentistry and oral healthcare).

1. To develop appropriate eating habits, including but not limited to choosing healthy food and eating less hard food.
2. To keep good oral hygiene habits, such as rinsing mouth after each diet and using household protective articles including dental flushing device, dental floss, mouthwash.
3. To perform regular oral examination to evaluate new caries and oral soft tissue problems.
4. To intervene in irreversible diseases such as nocturnal molars and traumatic occlusion as early as conditions allow.
5. To timely repair the missing teeth to ensure the integrity of dentition and protect the remaining healthy teeth within the mouth.
6. To screen oral and maxillofacial tumors regardless of benign and malignant ones in the elderly, with an effective control of the development of dental neoplasia.
7. Doctors and patients of dental clinics need to establish goals and outline a good prognosis.
8. To store oral healthcare guidebooks in community service center or set up an oral lecture hall to invite doctors to publicize oral healthcare knowledge and serve residents.

## 7. Concluding remarks and prospects

Due to the distinctive process of tooth development, the formation of hard tooth tissue is transient. Both the dentin-forming odontoblasts and the enamel-forming ameloblasts belong to highly differentiated cell populations, which are difficult to separate and culture. Although studies focusing on tooth aging tends to increase in recent years, current biotechnologies remain unable to successfully achieve functional regeneration of tooth hard tissue, necessitating continued search for materials to aid in hard tissue replacement and tooth implant. At present, the research of tooth aging is far behind that of the vast majority of other organs, such as bone, muscle, skin, liver, lung and blood.

The future prospects in human dental aging intervention can be largely summarized as follows (in the order of technical priority and scientific significance).

1. Dental pulp regeneration: at present, we mainly focus on pulp regeneration of young permanent teeth with partial pulp necrosis. Although advances have been made in regenerating materials, cells and cell-derived factors, there is still a long way to go before functional pulp tissue is regenerated, with regeneration of the pulp of the elderly still challenging.
2. Enamel regeneration and repair: the elderly are often confronted with problems of enamel structure change and repair, but enamel regeneration has not been made possible to date, whereas major progress is basically limited to selection and improvement of materials.
3. Systematic study of the difference between the internal nervous system of teeth, the peripheral nervous system, and the central nervous system (CNS) of other organs may help block and rebuild the link between the dental nerve and the CNS.
4. Aged pulp is always accompanied by an increased accumulation of fat and fibrous cells. The systematic comparison between the fat cells in the pulp and the fat in the abdomen of the human body may provide a solution to achieve a scientific weight loss of the pulp tissue.
5. Tooth aging related model: conventional aging models of DPSCs, or those extracted from elderly teeth, have proved feasible for screening drugs or related molecular mechanisms study, but the real *in vivo* model has not yet been developed. *In vivo* models of non-human primates may represent an optimal alternative, in spite of high costs.
6. Non-invasive diagnosis of teeth: with the advancement of modern optics and microdetection technology, related detection equipment is likely to achieve a breakthrough in the next decade. It is imaginable to determine tooth aging quickly, accurately, and noninvasively at home in future.
7. Small molecule compounds: can not only promote dentin regeneration, but even reverse the aging of pulp stem cells or odontoblasts in the near future, making tooth damage repair more efficient and straightforward.

Anyway, we firmly believe that the future antiaging research of human teeth will make significant and continued progress, according to the advance rate of aging-related studies performed for the vast majority of other organs, eventually benefiting the whole mankind.

## References

[1] Preshaw PM, Henne K, Taylor JJ, Valentine RA, Conrads G (2017). Age-related changes in immune function (immune senescence) in caries and periodontal diseases: a systematic review. J Clin Periodontol, 44 Suppl 18:S153-S177. 28266110 10.1111/jcpe.12675

[2] Franceschi C, Garagnani P, Parini P, Giuliani C, Santoro A (2018). Inflammaging: a new immune-metabolic viewpoint for age-related diseases. Nat Rev Endocrinol, 14:576-590. 30046148 10.1038/s41574-018-0059-4

[3] Hayflick L, Moorhead PS (1961). The serial cultivation of human diploid cell strains. Exp Cell Res, 25:585-621. 13905658 10.1016/0014-4827(61)90192-6

[4] Fridlyanskaya I, Alekseenko L, Nikolsky N (2015). Senescence as a general cellular response to stress: A mini-review. Experimental Gerontology, 72:124-128. 26435346 10.1016/j.exger.2015.09.021

[5] McHugh D, Gil J (2018). Senescence and aging: Causes, consequences, and therapeutic avenues. J Cell Biol, 217:65-77. 29114066 10.1083/jcb.201708092 PMC5748990

[6] Ogrodnik M, Evans SA, Fielder E, Victorelli S, Kruger P, Salmonowicz H, et al. (2021). Whole-body senescent cell clearance alleviates age-related brain inflammation and cognitive impairment in mice. Aging Cell, 20:e13296. 33470505 10.1111/acel.13296 PMC7884042

[7] Gasek NS, Kuchel GA, Kirkland JL, Xu M (2021). Strategies for Targeting Senescent Cells in Human Disease. Nat Aging, 1:870-879. 34841261 10.1038/s43587-021-00121-8 PMC8612694

[8] Song S, Lam EW, Tchkonia T, Kirkland JL, Sun Y (2020). Senescent Cells: Emerging Targets for Human Aging and Age-Related Diseases. Trends Biochem Sci, 45:578-592. 32531228 10.1016/j.tibs.2020.03.008 PMC7649645

[9] Hernandez-Segura A, Nehme J, Demaria M (2018). Hallmarks of Cellular Senescence. Trends in Cell Biology, 28:436-453. 29477613 10.1016/j.tcb.2018.02.001

[10] Hernandez-Segura A, Brandenburg S, Demaria M (2018). Induction and Validation of Cellular Senescence in Primary Human Cells. [J] Vis Exp. 10.3791/57782 PMC6101959 29985363

[11] Dimri GP, Lee XH, Basile G, Acosta M, Scott C, Roskelley C, et al. (1995). A Biomarker That Identifies Senescent Human-Cells in Culture and in Aging Skin in-Vivo. Proceedings of the National Academy of Sciences of the United States of America, 92:9363-9367. 7568133 10.1073/pnas.92.20.9363 PMC40985

[12] Lopez-Otin C, Blasco MA, Partridge L, Serrano M, Kroemer G (2013). The hallmarks of aging. Cell, 153:1194-1217. 23746838 10.1016/j.cell.2013.05.039 PMC3836174

[13] Martinez-Lopez N, Athonvarangkul D, Singh R (2015). Autophagy and aging. Adv Exp Med Biol, 847:73-87. 25916586 10.1007/978-1-4939-2404-2_3 PMC4644734

[14] Coppe JP, Patil CK, Rodier F, Sun Y, Munoz DP, Goldstein J, et al. (2008). Senescence-associated secretory phenotypes reveal cell-nonautonomous functions of oncogenic RAS and the p53 tumor suppressor. PLoS Biol, 6:2853-2868. 19053174 10.1371/journal.pbio.0060301 PMC2592359

[15] Childs BG, Baker DJ, Kirkland JL, Campisi J, van Deursen JM (2014). Senescence and apoptosis: dueling or complementary cell fates? EMBO Rep, 15:1139-1153. 25312810 10.15252/embr.201439245 PMC4253488

[16] Song S, Tchkonia T, Jiang J, Kirkland JL, Sun Y (2020). Targeting Senescent Cells for a Healthier Aging: Challenges and Opportunities. Adv Sci (Weinh), 7:2002611. 33304768 10.1002/advs.202002611 PMC7709980

[17] Coppe JP, Desprez PY, Krtolica A, Campisi J (2010). The senescence-associated secretory phenotype: the dark side of tumor suppression. Annu Rev Pathol, 5:99-118. 20078217 10.1146/annurev-pathol-121808-102144 PMC4166495

[18] Larsson C, Hansson EE, Sundquist K, Jakobsson U (2017). Chronic pain in older adults: prevalence, incidence, and risk factors. Scand J Rheumatol, 46:317-325. 27885914 10.1080/03009742.2016.1218543

[19] Astvaldsdottir A, Bostrom AM, Davidson T, Gabre P, Gahnberg L, Sandborgh Englund G, et al. (2018). Oral health and dental care of older persons-A systematic map of systematic reviews. Gerodontology, 35:290-304. 30129220 10.1111/ger.12368

[20] Kassebaum NJ, Bernabe E, Dahiya M, Bhandari B, Murray CJL, Marcenes W (2015). Global Burden of Untreated Caries: A Systematic Review and Metaregression. Journal of Dental Research, 94:650-658. 25740856 10.1177/0022034515573272

[21] Mitsiadis TA, Orsini G, Jimenez-Rojo L (2015). Stem cell-based approaches in dentistry. Eur Cell Mater, 30:248-257. 26562631 10.22203/ecm.v030a17

[22] Orsini G, Pagella P, Putignano A, Mitsiadis TA (2018). Novel Biological and Technological Platforms for Dental Clinical Use. Frontiers in Physiology, 9. 30135661 10.3389/fphys.2018.01102 PMC6092501

[23] Gronthos S, Mankani M, Brahim J, Robey PG, Shi S (2000). Postnatal human dental pulp stem cells (DPSCs) in vitro and in vivo. Proc Natl Acad Sci U S A, 97:13625-13630. 11087820 10.1073/pnas.240309797 PMC17626

[24] Bull FC, Al-Ansari SS, Biddle S, Borodulin K, Buman MP, Cardon G, et al. (2020). World Health Organization 2020 guidelines on physical activity and sedentary behaviour. Br J Sports Med, 54:1451-1462. 33239350 10.1136/bjsports-2020-102955 PMC7719906

[25] Ritter AV, Shugars DA, Bader JD (2010). Root caries risk indicators: a systematic review of risk models. Community Dent Oral Epidemiol, 38:383-397. 20545716 10.1111/j.1600-0528.2010.00551.x PMC2962697

[26] Anu V, Avanthika K, Angeline B, Rachel JB (2020). Oral healthcare needs of the institutionalized ageing population in Tamil Nadu. Natl Med J India, 33:83-85. 33753635 10.4103/0970-258X.310983

[27] Porceddu SV, Haddad RI (2017). Management of elderly patients with locoregionally confined head and neck cancer. Lancet Oncol, 18:e274-e283. 28456589 10.1016/S1470-2045(17)30229-2

[28] Smith BD, Smith GL, Hurria A, Hortobagyi GN, Buchholz TA (2009). Future of cancer incidence in the United States: burdens upon an aging, changing nation. J Clin Oncol, 27:2758-2765. 19403886 10.1200/JCO.2008.20.8983

[29] Metgud R, Anandani C, Singh K (2015). Estimation of salivary nitric oxide in oral precancer patients. Biotech Histochem, 90:302-308. 25831210 10.3109/10520295.2014.998282

[30] Wu Y, Zhang B, Huang Z, Ruan Y, Huang Z (2018). Study of surgical treatment for elderly patients with head and neck cancer. Int J Oral Maxillofac Surg, 47:824-829. 29429844 10.1016/j.ijom.2018.01.018

[31] Ulaganathan G, Babu SS, Senthilmoorthy M, Prasad V, Kalaiselvan S, Kumar RSA (2020). Retrospective Analysis of Oral and Maxillofacial Biopsies: An Institutional Study. J Pharm Bioallied Sci, 12:S468-S471. 33149507 10.4103/jpbs.JPBS_141_20 PMC7595515

[32] Kaplan I, Raiser V, Shuster A, Shlomi B, Rosenfeld E, Greenberg A, et al. (2019). Metastatic tumors in oral mucosa and jawbones: Unusual primary origins and unusual oral locations. Acta Histochem, 121:151448. 31570205 10.1016/j.acthis.2019.151448

[33] Schenkein HA, Loos BG (2013). Inflammatory mechanisms linking periodontal diseases to cardiovascular diseases. J Clin Periodontol, 40 Suppl 14:S51-69. 23627334 10.1111/jcpe.12060 PMC4554326

[34] Pietropaoli D, Del Pinto R, Ferri C, Wright JT Jr., Giannoni M, Ortu E, et al. (2018). Poor Oral Health and Blood Pressure Control Among US Hypertensive Adults. Hypertension, 72:1365-1373. 30540406 10.1161/HYPERTENSIONAHA.118.11528

[35] Lopez-de-Andres A, Vazquez-Vazquez L, Martinez-Huedo MA, Hernandez-Barrera V, Jimenez-Trujillo I, Tapias-Ledesma MA, et al. (2018). Is COPD associated with periodontal disease? A population-based study in Spain. Int J Chron Obstruct Pulmon Dis, 13:3435-3445. 30425473 10.2147/COPD.S174898 PMC6203114

[36] Shen TC, Chang PY, Lin CL, Chen CH, Tu CY, Hsia TC, et al. (2016). Periodontal Treatment Reduces Risk of Adverse Respiratory Events in Patients With Chronic Obstructive Pulmonary Disease: A Propensity-Matched Cohort Study. Medicine (Baltimore), 95:e3735. 27196497 10.1097/MD.0000000000003735 PMC4902439

[37] Qian Y, Yuan W, Mei N, Wu J, Xu Q, Lu H, et al. (2020). Periodontitis increases the risk of respiratory disease mortality in older patients. Exp Gerontol, 133:110878. 32061644 10.1016/j.exger.2020.110878

[38] Genco RJ, Sanz M (2020). Clinical and public health implications of periodontal and systemic diseases: An overview. Periodontol 2000, 83:7-13. 10.1111/prd.12344 32385880

[39] Chhibber-Goel J, Singhal V, Bhowmik D, Vivek R, Parakh N, Bhargava B, et al. (2016). Linkages between oral commensal bacteria and atherosclerotic plaques in coronary artery disease patients. NPJ Biofilms Microbiomes, 2:7. 28649401 10.1038/s41522-016-0009-7 PMC5460270

[40] Kavarthapu A, Gurumoorthy K (2021). Linking chronic periodontitis and oral cancer: A review. Oral Oncol, 121:105375. 34140233 10.1016/j.oraloncology.2021.105375

[41] Karmakar S, Kar A, Thakur S, Rao VUS (2020). Periodontitis and oral Cancer-A striking link. Oral Oncol, 106:104630. 32147310 10.1016/j.oraloncology.2020.104630

[42] Lee WH, Chen HM, Yang SF, Liang C, Peng CY, Lin FM, et al. (2017). Bacterial alterations in salivary microbiota and their association in oral cancer. Sci Rep, 7:16540. 29184122 10.1038/s41598-017-16418-x PMC5705712

[43] Wu H, Qiu W, Zhu X, Li X, Xie Z, Carreras I, et al. (2022). The Periodontal Pathogen Fusobacterium nucleatum Exacerbates Alzheimer's Pathogenesis via Specific Pathways. Front Aging Neurosci, 14:912709. 35813949 10.3389/fnagi.2022.912709 PMC9260256

[44] Yang B, Tao B, Yin Q, Chai Z, Xu L, Zhao Q, et al. (2021). Associations Between Oral Health Status, Perceived Stress, and Neuropsychiatric Symptoms Among Community Individuals With Alzheimer's Disease: A Mediation Analysis. Front Aging Neurosci, 13:801209. 35082659 10.3389/fnagi.2021.801209 PMC8786079

[45] Komine-Aizawa S, Aizawa S, Hayakawa S (2019). Periodontal diseases and adverse pregnancy outcomes. J Obstet Gynaecol Res, 45:5-12. 30094895 10.1111/jog.13782

[46] Mahendra J, Mahendra L, Sharma V, Alamoudi A, Bahammam HA, Mugri MH, et al. (2022). Red-Complex Bacterial Levels in Pregnant Women With Preeclampsia and Chronic Periodontitis. Int Dent J. 10.1016/j.identj.2022.10.003 PMC10350591 36411127

[47] Chattopadhyay I, Lu W, Manikam R, Malarvili MB, Ambati RR, Gundamaraju R (2022). Can metagenomics unravel the impact of oral bacteriome in human diseases? Biotechnol Genet Eng Rev:1-33. 10.1080/02648725.2022.2102877 35861776

[48] Zhong J, Shibata Y (2022). The structural motifs of mineralized hard tissues from nano- to mesoscale: A future perspective for material science. Jpn Dent Sci Rev, 58:348-356. 36404956 10.1016/j.jdsr.2022.11.001 PMC9672955

[49] Qiu YN, Liu WM, Li TS, Liu LZ (2003). [Comparative study of friction and wear behavior of different human enamel in vitro]. Zhonghua Kou Qiang Yi Xue Za Zhi, 38:213-216. 12887802

[50] Weatherell JA, Robinson C, Hallsworth AS (1974). Variations in the chemical composition of human enamel. J Dent Res, 53:180-192. 4591966 10.1177/00220345740530020501

[51] Chemelo VDS, Ne YGS, Frazao DR, de Souza-Rodrigues RD, Fagundes NCF, Magno MB, et al. (2020). Is There Association Between Stress and Bruxism? A Systematic Review and Meta-Analysis. Front Neurol, 11:590779. 33424744 10.3389/fneur.2020.590779 PMC7793806

[52] Iezzi I, Pagella P, Mattioli-Belmonte M, Mitsiadis TA (2019). The effects of ageing on dental pulp stem cells, the tooth longevity elixir. Eur Cell Mater, 37:175-185. 30805914 10.22203/eCM.v037a11

[53] Morse DR, Rabinowitz H (1990). A unified theory of aging. Int J Psychosom, 37:5-24. 2246103

[54] Carvalho TS, Lussi A (2015). Susceptibility of enamel to initial erosion in relation to tooth type, tooth surface and enamel depth. Caries Res, 49:109-115. 25592786 10.1159/000369104

[55] Tilley LP, Weitz J (1977). Pharmacologic and other forms of medical therapy in feline cardiac disease. Vet Clin North Am, 7:415-428. 325874 10.1016/s0091-0279(77)50039-1

[56] Park S, Wang DH, Zhang D, Romberg E, Arola D (2008). Mechanical properties of human enamel as a function of age and location in the tooth. J Mater Sci Mater Med, 19:2317-2324. 18157510 10.1007/s10856-007-3340-y

[57] Yan W, Renteria C, Huang Y, Arola DD (2021). A machine learning approach to investigate the materials science of enamel aging. Dent Mater, 37:1761-1771. 34625295 10.1016/j.dental.2021.09.006

[58] Kinney JH, Nalla RK, Pople JA, Breunig TM, Ritchie RO (2005). Age-related transparent root dentin: mineral concentration, crystallite size, and mechanical properties. Biomaterials, 26:3363-3376. 15603832 10.1016/j.biomaterials.2004.09.004

[59] Porter AE, Nalla RK, Minor A, Jinschek JR, Kisielowski C, Radmilovic V, et al. (2005). A transmission electron microscopy study of mineralization in age-induced transparent dentin. Biomaterials, 26:7650-7660. 16005961 10.1016/j.biomaterials.2005.05.059

[60] Ivancik J, Majd H, Bajaj D, Romberg E, Arola D (2012). Contributions of aging to the fatigue crack growth resistance of human dentin. Acta Biomater, 8:2737-2746. 22484693 10.1016/j.actbio.2012.03.046 PMC3367091

[61] Yahyazadehfar M, Ivancik J, Majd H, An B, Zhang D, Arola D (2014). On the Mechanics of Fatigue and Fracture in Teeth. Appl Mech Rev, 66:0308031-3080319. 25516632 10.1115/1.4027431 PMC4240032

[62] Shinno Y, Ishimoto T, Saito M, Uemura R, Arino M, Marumo K, et al. (2016). Comprehensive analyses of how tubule occlusion and advanced glycation end-products diminish strength of aged dentin. Sci Rep, 6:19849. 26797297 10.1038/srep19849 PMC4726429

[63] Ryou H, Romberg E, Pashley DH, Tay FR, Arola D (2015). Importance of age on the dynamic mechanical behavior of intertubular and peritubular dentin. J Mech Behav Biomed Mater, 42:229-242. 25498296 10.1016/j.jmbbm.2014.11.021 PMC4290863

[64] Yahyazadehfar M, Zhang D, Arola D (2016). On the importance of aging to the crack growth resistance of human enamel. Acta Biomater, 32:264-274. 26747980 10.1016/j.actbio.2015.12.038 PMC4754146

[65] Porter ML, Munchow EA, Albuquerque MT, Spolnik KJ, Hara AT, Bottino MC (2016). Effects of Novel 3-dimensional Antibiotic-containing Electrospun Scaffolds on Dentin Discoloration. J Endod, 42:106-112. 26602451 10.1016/j.joen.2015.09.013 PMC4913479

[66] Suominen I, Andersson MA, Andersson MC, Hallaksela AM, Kampfer P, Rainey FA, et al. (2001). Toxic Bacillus pumilus from indoor air, recycled paper pulp, Norway spruce, food poisoning outbreaks and clinical samples. Syst Appl Microbiol, 24:267-276. 11518331 10.1078/0723-2020-00025

[67] Masuda K, Han X, Kato H, Sato H, Zhang Y, Sun X, et al. (2021). Dental Pulp-Derived Mesenchymal Stem Cells for Modeling Genetic Disorders. Int J Mol Sci, 22. 33668763 10.3390/ijms22052269 PMC7956585

[68] Seltzer S (1972). Classification of pulpal pathosis. Oral Surg Oral Med Oral Pathol, 34:269-287. 4556806 10.1016/0030-4220(72)90419-7

[69] Ge ZP, Yang P, Li G, Zhang JZ, Ma XC (2016). Age estimation based on pulp cavity/chamber volume of 13 types of tooth from cone beam computed tomography images. Int J Legal Med, 130:1159-1167. 27221534 10.1007/s00414-016-1384-6

[70] Porto LV, Celestino da Silva Neto J, Anjos Pontual AD, Catunda RQ (2015). Evaluation of volumetric changes of teeth in a Brazilian population by using cone beam computed tomography. J Forensic Leg Med, 36:4-9. 26320003 10.1016/j.jflm.2015.07.007

[71] Pinchi V, Pradella F, Buti J, Baldinotti C, Focardi M, Norelli GA (2015). A new age estimation procedure based on the 3D CBCT study of the pulp cavity and hard tissues of the teeth for forensic purposes: A pilot study. J Forensic Leg Med, 36:150-157. 26458182 10.1016/j.jflm.2015.09.015

[72] Gulsahi A, Cebeci AI, Ozden S (2009). A radiographic assessment of the prevalence of pulp stones in a group of Turkish dental patients. Int Endod J, 42:735-739. 19549152 10.1111/j.1365-2591.2009.01580.x

[73] Schaffner M, Stich H, Lussi A (2014). [Denticles: dental pulp calculi]. Swiss Dent J, 124:416-417. 24805138 10.61872/sdj-2014-04-02

[74] Kannan S, Kannepady SK, Muthu K, Jeevan MB, Thapasum A (2015). Radiographic assessment of the prevalence of pulp stones in Malaysians. J Endod, 41:333-337. 25476972 10.1016/j.joen.2014.10.015

[75] Bains SK, Bhatia A, Singh HP, Biswal SS, Kanth S, Nalla S (2014). Prevalence of coronal pulp stones and its relation with systemic disorders in northern Indian central punjabi population. ISRN Dent, 2014:617590. 24944821 10.1155/2014/617590 PMC4040191

[76] Sisman Y, Aktan AM, Tarim-Ertas E, Ciftci ME, Sekerci AE (2012). The prevalence of pulp stones in a Turkish population. A radiographic survey. Med Oral Patol Oral Cir Bucal, 17:e212-217. 22143688 10.4317/medoral.17400 PMC3448315

[77] Ketterl W (1983). Age-induced changes in the teeth and their attachment apparatus. Int Dent J, 33:262-271. 6579031

[78] Weerakoon AT, Meyers IA, Thomson DH, Cooper C, Ford PJ, Symons AL (2022). Coronal dentin differs between young and mature adult humans: A systematic review. Arch Oral Biol, 144:105553. 36182707 10.1016/j.archoralbio.2022.105553

[79] Nalbandian J, Gonzales F, Sognnaes RF (1960). Sclerotic age changes in root dentin of human teeth as observed by optical, electron, and x-ray microscopy. J Dent Res, 39:598-607. 14425932 10.1177/00220345600390032101

[80] Larmas MA, Sandor GK (2013). Solid nomenclature: the bedrock of science. Similarities and dissimilarities in phenomena and cells of tooth and bone ontogeny. Anat Rec (Hoboken), 296:564-567. 23420639 10.1002/ar.22671

[81] Couve E, Osorio R, Schmachtenberg O (2013). The amazing odontoblast: activity, autophagy, and aging. J Dent Res, 92:765-772. 23803461 10.1177/0022034513495874

[82] Nelson BL, Thompson LDR (2019). A Rainbow of Colors and Spectrum of Textures: An Approach to Oral Mucosal Entities. Head Neck Pathol, 13:1-3. 30693461 10.1007/s12105-019-01007-3 PMC6405790

[83] Zavala-Alonso V, Martinez-Castanon GA, Patino-Marin N, Terrones H, Anusavice K, Loyola-Rodriguez JP (2010). Characterization of healthy and fluorotic enamel by atomic force microscopy. Microsc Microanal, 16:531-536. 20813079 10.1017/S1431927610093748

[84] Nassar Y, Brizuela M.2022. The Role Of Fluoride On Caries Prevention. In StatPearls. Treasure Island (FL). 36508516

[85] Chen Y, Wu R, Shen L, Yang Y, Wang G, Yang B (2022). The multi-scale meso-mechanics model of viscoelastic dentin. J Mech Behav Biomed Mater, 136:105525. 36302275 10.1016/j.jmbbm.2022.105525

[86] Song YL, Wang CN, Fan MW, Su B, Bian Z (2008). Dentin phosphoprotein frameshift mutations in hereditary dentin disorders and their variation patterns in normal human population. J Med Genet, 45:457-464. 18456718 10.1136/jmg.2007.056911

[87] Song Y, Wang C, Peng B, Ye X, Zhao G, Fan M, et al. (2006). Phenotypes and genotypes in 2 DGI families with different DSPP mutations. Oral Surg Oral Med Oral Pathol Oral Radiol Endod, 102:360-374. 16920545 10.1016/j.tripleo.2005.06.020

[88] Lindau BM, Dietz W, Hoyer I, Lundgren T, Storhaug K, Noren JG (1999). Morphology of dental enamel and dentine-enamel junction in osteogenesis imperfecta. Int J Paediatr Dent, 9:13-21. 10336712 10.1046/j.1365-263x.1999.00101.x

[89] Gallusi G, Libonati A, Campanella V (2006). SEM-morphology in dentinogenesis imperfecta type II: microscopic anatomy and efficacy of a dentine bonding system. Eur J Paediatr Dent, 7:9-17. 16646639

[90] Nazir MA (2017). Prevalence of periodontal disease, its association with systemic diseases and prevention. Int J Health Sci (Qassim), 11:72-80. 28539867 PMC5426403

[91] Chen FM, Gao LN, Tian BM, Zhang XY, Zhang YJ, Dong GY, et al. (2016). Treatment of periodontal intrabony defects using autologous periodontal ligament stem cells: a randomized clinical trial. Stem Cell Res Ther, 7:33. 26895633 10.1186/s13287-016-0288-1 PMC4761216

[92] Balhaddad AA, Kansara AA, Hidan D, Weir MD, Xu HHK, Melo MAS (2019). Toward dental caries: Exploring nanoparticle-based platforms and calcium phosphate compounds for dental restorative materials. Bioact Mater, 4:43-55. 30582079 10.1016/j.bioactmat.2018.12.002 PMC6299130

[93] Azaryan E, Emadian Razavi F, Hanafi-Bojd MY, Alemzadeh E, Naseri M (2022). Dentin Regeneration based on Tooth Tissue Engineering: A review. Biotechnol Prog:e3319. 10.1002/btpr.3319 36522133

[94] Maeda H (2020). Aging and Senescence of Dental Pulp and Hard Tissues of the Tooth. Front Cell Dev Biol, 8:605996. 33330507 10.3389/fcell.2020.605996 PMC7734349

[95] Thesleff I, Keranen S, Jernvall J (2001). Enamel knots as signaling centers linking tooth morphogenesis and odontoblast differentiation. Adv Dent Res, 15:14-18. 12640732 10.1177/08959374010150010401

[96] Nanci A (2018). Ten Cate’s Oral Histology. 9th Edition. St. Louis, MO: Elsevier.

[97] Mahdee A, Eastham J, Whitworth JM, Gillespie JI (2019). Evidence for changing nerve growth factor signalling mechanisms during development, maturation and ageing in the rat molar pulp. Int Endod J, 52:211-222. 30099752 10.1111/iej.12997

[98] Ersahan S, Sabuncuoglu FA (2018). Effect of age on pulpal blood flow in human teeth during orthodontic movement. J Oral Sci, 60:446-452. 30249934 10.2334/josnusd.17-0316

[99] Murray PE, Stanley HR, Matthews JB, Sloan AJ, Smith AJ (2002). Age-related odontometric changes of human teeth. Oral Surg Oral Med Oral Pathol Oral Radiol Endod, 93:474-482. 12029288 10.1067/moe.2002.120974

[100] Jafarzadeh H, Abbott PV (2010). Review of pulp sensibility tests. Part I: general information and thermal tests. Int Endod J, 43:738-762. 20609022 10.1111/j.1365-2591.2010.01754.x

[101] Krasner P, Rankow HJ (2004). Anatomy of the pulp-chamber floor. J Endod, 30:5-16. 14760900 10.1097/00004770-200401000-00002

[102] Bernick S, Nedelman C (1975). Effect of aging on the human pulp. J Endod, 1:88-94. 1061788 10.1016/S0099-2399(75)80024-0

[103] Oreski NP, Celebic A, Petricevic N (2017). Assessment of esthetic characteristics of the teeth and surrounding anatomical structures. Acta Stomatol Croat, 51:22-32. 28740267 10.15644/asc51/1/3 PMC5506250

[104] Saad AY (1997). Regressive changes of the dental pulp complex in retained primary molars with congenitally missing successor teeth. J Clin Pediatr Dent, 22:63-67. 9643208

[105] Varalli A, Desideri J, David-Elbiali M, Goude G, Honegger M, Besse M (2021). Bronze Age innovations and impact on human diet: A multi-isotopic and multi-proxy study of western Switzerland. PLoS One, 16:e0245726. 33503025 10.1371/journal.pone.0245726 PMC7840060

[106] Morse DR (1991). Age-related changes of the dental pulp complex and their relationship to systemic aging. Oral Surg Oral Med Oral Pathol, 72:721-745. 1812456 10.1016/0030-4220(91)90019-9

[107] Couve E, Osorio R, Schmachtenberg O (2012). Mitochondrial autophagy and lipofuscin accumulation in aging odontoblasts. J Dent Res, 91:696-701. 22622661 10.1177/0022034512449347

[108] Shi S, Gronthos S (2003). Perivascular niche of postnatal mesenchymal stem cells in human bone marrow and dental pulp. J Bone Miner Res, 18:696-704. 12674330 10.1359/jbmr.2003.18.4.696

[109] Zhao H, Feng J, Seidel K, Shi S, Klein O, Sharpe P, et al. (2018). Secretion of Shh by a Neurovascular Bundle Niche Supports Mesenchymal Stem Cell Homeostasis in the Adult Mouse Incisor. Cell Stem Cell, 23:147. 29979989 10.1016/j.stem.2018.05.023 PMC6039109

[110] Carr MJ, Toma JS, Johnston APW, Steadman PE, Yuzwa SA, Mahmud N, et al. (2019). Mesenchymal Precursor Cells in Adult Nerves Contribute to Mammalian Tissue Repair and Regeneration. Cell Stem Cell, 24:240-256 e249. 30503141 10.1016/j.stem.2018.10.024

[111] Janebodin K, Horst OV, Ieronimakis N, Balasundaram G, Reesukumal K, Pratumvinit B, et al. (2011). Isolation and characterization of neural crest-derived stem cells from dental pulp of neonatal mice. PLoS One, 6:e27526. 22087335 10.1371/journal.pone.0027526 PMC3210810

[112] Kaukua N, Shahidi MK, Konstantinidou C, Dyachuk V, Kaucka M, Furlan A, et al. (2014). Glial origin of mesenchymal stem cells in a tooth model system. Nature, 513:551-554. 25079316 10.1038/nature13536

[113] Amoh Y, Li L, Campillo R, Kawahara K, Katsuoka K, Penman S, et al. (2005). Implanted hair follicle stem cells form Schwann cells that support repair of severed peripheral nerves. Proc Natl Acad Sci U S A, 102:17734-17738. 16314569 10.1073/pnas.0508440102 PMC1308908

[114] Zhan C, Huang M, Yang X, Hou J (2021). Dental nerves: a neglected mediator of pulpitis. Int Endod J, 54:85-99. 32880979 10.1111/iej.13400

[115] Aguilar P, Linsuwanont P (2011). Vital pulp therapy in vital permanent teeth with cariously exposed pulp: a systematic review. J Endod, 37:581-587. 21496652 10.1016/j.joen.2010.12.004

[116] Parirokh M, Torabinejad M (2010). Mineral trioxide aggregate: a comprehensive literature review--Part III: Clinical applications, drawbacks, and mechanism of action. J Endod, 36:400-413. 20171353 10.1016/j.joen.2009.09.009

[117] Paula A, Laranjo M, Marto CM, Abrantes AM, Casalta-Lopes J, Goncalves AC, et al. (2019). Biodentine() Boosts, WhiteProRoot((R))MTA Increases and Life((R)) Suppresses Odontoblast Activity. Materials (Basel), 12. 30978943 10.3390/ma12071184 PMC6479701

[118] Ong TK, Lim GS, Singh M, Fial AV (2020). Quantitative Assessment of Root Development after Regenerative Endodontic Therapy: A Systematic Review and Meta-Analysis. J Endod, 46:1856-1866 e1852. 32827507 10.1016/j.joen.2020.08.016

[119] Murray PE, Garcia-Godoy F, Hargreaves KM (2007). Regenerative endodontics: a review of current status and a call for action. J Endod, 33:377-390. 17368324 10.1016/j.joen.2006.09.013

[120] Schmalz G, Widbiller M, Galler KM (2020). Clinical Perspectives of Pulp Regeneration. J Endod, 46:S161-S174. 32950188 10.1016/j.joen.2020.06.037

[121] Alongi DJ, Yamaza T, Song Y, Fouad AF, Romberg EE, Shi S, et al. (2010). Stem/progenitor cells from inflamed human dental pulp retain tissue regeneration potential. Regen Med, 5:617-631. 20465527 10.2217/rme.10.30 PMC3035701

[122] Di Micco R, Krizhanovsky V, Baker D, d'Adda di Fagagna F (2021). Cellular senescence in ageing: from mechanisms to therapeutic opportunities. Nat Rev Mol Cell Biol, 22:75-95. 33328614 10.1038/s41580-020-00314-w PMC8344376

[123] Yang RL, Huang HM, Han CS, Cui SJ, Zhou YK, Zhou YH (2021). Serine Metabolism Controls Dental Pulp Stem Cell Aging by Regulating the DNA Methylation of p16. J Dent Res, 100:90-97. 32940141 10.1177/0022034520958374

[124] Liang H, Li W, Yang H, Cao Y, Ge L, Shi R, et al. (2020). FAM96B inhibits the senescence of dental pulp stem cells. Cell Biol Int, 44:1193-1203. 32039527 10.1002/cbin.11319

[125] Macrin D, Alghadeer A, Zhao YT, Miklas JW, Hussein AM, Detraux D, et al. (2019). Metabolism as an early predictor of DPSCs aging. Scientific Reports, 9. 30778087 10.1038/s41598-018-37489-4 PMC6379364

[126] Yi Q, Liu O, Yan F, Lin X, Diao S, Wang L, et al. (2017). Analysis of Senescence-Related Differentiation Potentials and Gene Expression Profiles in Human Dental Pulp Stem Cells. Cells Tissues Organs, 203:1-11. 27627434 10.1159/000448026

[127] Iezzi I, Cerqueni G, Licini C, Lucarini G, Mattioli Belmonte M (2019). Dental pulp stem cells senescence and regenerative potential relationship. J Cell Physiol, 234:7186-7197. 30362542 10.1002/jcp.27472

[128] Narayanan K, Ramachandran A, Hao J, He G, Park KW, Cho M, et al. (2003). Dual functional roles of dentin matrix protein 1. Implications in biomineralization and gene transcription by activation of intracellular Ca2+ store. J Biol Chem, 278:17500-17508. 12615915 10.1074/jbc.M212700200

[129] Qin C, Brunn JC, Cook RG, Orkiszewski RS, Malone JP, Veis A, et al. (2003). Evidence for the proteolytic processing of dentin matrix protein 1. Identification and characterization of processed fragments and cleavage sites. J Biol Chem, 278:34700-34708. 12813042 10.1074/jbc.M305315200

[130] Piattelli A, Trisi P (1993). Pulp obliteration: a histological study. J Endod, 19:252-254. 8360605 10.1016/S0099-2399(06)81303-8

[131] Bressan E, Ferroni L, Gardin C, Pinton P, Stellini E, Botticelli D, et al. (2012). Donor age-related biological properties of human dental pulp stem cells change in nanostructured scaffolds. PLoS One, 7:e49146. 23209565 10.1371/journal.pone.0049146 PMC3509126

[132] Hannas AR, Pereira JC, Granjeiro JM, Tjaderhane L (2007). The role of matrix metalloproteinases in the oral environment. Acta Odontol Scand, 65:1-13. 17354089 10.1080/00016350600963640

[133] Shin SJ, Lee JI, Baek SH, Lim SS (2002). Tissue levels of matrix metalloproteinases in pulps and periapical lesions. J Endod, 28:313-315. 12043871 10.1097/00004770-200204000-00013

[134] Goda S, Kato Y, Domae E, Hayashi H, Tani-Ishii N, Iida J, et al. (2015). Effects of JNK1/2 on the inflammation cytokine TNF-alpha-enhanced production of MMP-3 in human dental pulp fibroblast-like cells. Int Endod J, 48:1122-1128. 25393585 10.1111/iej.12411

[135] Pezelj-Ribaric S, Anic I, Brekalo I, Miletic I, Hasan M, Simunovic-Soskic M (2002). Detection of tumor necrosis factor alpha in normal and inflamed human dental pulps. Arch Med Res, 33:482-484. 12459320 10.1016/s0188-4409(02)00396-x

[136] Liu H, Li W, Shi ST, Habelitz S, Gao C, DenBesten P (2005). MEPE is downregulated as dental pulp stem cells differentiate. Archives of Oral Biology, 50:923-928. 16183369 10.1016/j.archoralbio.2005.03.003

[137] Lee YH, Kim GE, Cho HJ, Yu MK, Bhattarai G, Lee NH, et al. (2013). Aging of In Vitro Pulp Illustrates Change of Inflammation and Dentinogenesis. Journal of Endodontics, 39:340-345. 23402504 10.1016/j.joen.2012.10.031

[138] Vollset SE, Goren E, Yuan CW, Cao J, Smith AE, Hsiao T, et al. (2020). Fertility, mortality, migration, and population scenarios for 195 countries and territories from 2017 to 2100: a forecasting analysis for the Global Burden of Disease Study. Lancet, 396:1285-1306. 32679112 10.1016/S0140-6736(20)30677-2 PMC7561721

[139] Glick M, Williams DM, Kleinman DV, Vujicic M, Watt RG, Weyant RJ (2016). A new definition for oral health developed by the FDI World Dental Federation opens the door to a universal definition of oral health. Int Dent J, 66:322-324. 27885673 10.1111/idj.12294 PMC9376665

[140] Peres MA, Macpherson LMD, Weyant RJ, Daly B, Venturelli R, Mathur MR, et al. (2019). Oral diseases: a global public health challenge. Lancet, 394:249-260. 31327369 10.1016/S0140-6736(19)31146-8

[141] Kassebaum NJ, Bernabe E, Dahiya M, Bhandari B, Murray CJ, Marcenes W (2015). Global burden of untreated caries: a systematic review and metaregression. J Dent Res, 94:650-658. 25740856 10.1177/0022034515573272

[142] Kassebaum NJ, Bernabe E, Dahiya M, Bhandari B, Murray CJ, Marcenes W (2014). Global burden of severe periodontitis in 1990-2010: a systematic review and meta-regression. J Dent Res, 93:1045-1053. 25261053 10.1177/0022034514552491 PMC4293771

[143] Huang G, Cao G (2022). Tooth Loss Trajectories and Their Association with Functional Disability among Older Chinese Adults: Results from the Chinese Longitudinal Healthy Longevity Survey. J Evid Based Dent Pract, 22:101771. 36494112 10.1016/j.jebdp.2022.101771

[144] Guilbert JJ (2006). The World Health Report 2006: working together for health. Educ Health (Abingdon), 19:385-387. 17178522 10.1080/13576280600937911

[145] Saari P, Heikkinen E, Sakari-Rantala R, Rantanen T (2007). Fall-related injuries among initially 75- and 80-year old people during a 10-year follow-up. Arch Gerontol Geriatr, 45:207-215. 17184857 10.1016/j.archger.2006.10.012

[146] Alexander BH, Rivara FP, Wolf ME (1992). The cost and frequency of hospitalization for fall-related injuries in older adults. Am J Public Health, 82:1020-1023. 1609903 10.2105/ajph.82.7.1020 PMC1694056

[147] Bergen G, Stevens MR, Burns ER (2016). Falls and Fall Injuries Among Adults Aged >/=65 Years - United States, 2014. MMWR Morb Mortal Wkly Rep, 65:993-998. 27656914 10.15585/mmwr.mm6537a2

[148] Razak PA, Richard KM, Thankachan RP, Hafiz KA, Kumar KN, Sameer KM (2014). Geriatric oral health: a review article. J Int Oral Health, 6:110-116. 25628498 PMC4295446

[149] Rutger Persson G (2012). Rheumatoid arthritis and periodontitis - inflammatory and infectious connections. Review of the literature. J Oral Microbiol, 4. 10.3402/jom.v4i0.11829 PMC3280043 22347541

[150] Sgolastra F, Petrucci A, Severino M, Gatto R, Monaco A (2013). Lasers for the treatment of dentin hypersensitivity: a meta-analysis. J Dent Res, 92:492-499. 23609160 10.1177/0022034513487212

[151] Ipci SD, Cakar G, Kuru B, Yilmaz S (2009). Clinical evaluation of lasers and sodium fluoride gel in the treatment of dentine hypersensitivity. Photomed Laser Surg, 27:85-91. 19182972 10.1089/pho.2008.2263

[152] Suri I, Singh P, Shakir QJ, Shetty A, Bapat R, Thakur R (2016). A comparative evaluation to assess the efficacy of 5% sodium fluoride varnish and diode laser and their combined application in the treatment of dentin hypersensitivity. J Indian Soc Periodontol, 20:307-314. 27563205 10.4103/0972-124X.181243 PMC4976552

[153] Mittal A, Nichani AS, Venugopal R, Rajani V (2014). The effect of various ultrasonic and hand instruments on the root surfaces of human single rooted teeth: A Planimetric and Profilometric study. J Indian Soc Periodontol, 18:710-717. 25624626 10.4103/0972-124X.147405 PMC4296454

[154] Foroutan T, Amid R, Karimi MR (2013). Comparison of Manual Tools, Ultrasonic and Erbium-Doped Yttrium Aluminum Garnet (Er:YAG) Laser on the Debridement Effect of the Surface of the Root of Teeth Suffering from Periodontitis. J Lasers Med Sci, 4:199-205. 25606330 PMC4282002

[155] Santin GC, Oliveira DS, Galo R, Borsatto MC, Corona SA (2014). Antimicrobial photodynamic therapy and dental plaque: a systematic review of the literature. ScientificWorldJournal, 2014:824538. 25379545 10.1155/2014/824538 PMC4212597

[156] Larsson L, Decker AM, Nibali L, Pilipchuk SP, Berglundh T, Giannobile WV (2016). Regenerative Medicine for Periodontal and Peri-implant Diseases. J Dent Res, 95:255-266. 26608580 10.1177/0022034515618887 PMC4766955

[157] Goncalves F, de Moraes MS, Ferreira LB, Carreira AC, Kossugue PM, Boaro LC, et al. (2016). Combination of Bioactive Polymeric Membranes and Stem Cells for Periodontal Regeneration: In Vitro and In Vivo Analyses. PLoS One, 11:e0152412. 27031990 10.1371/journal.pone.0152412 PMC4816539

[158] Zhang Y, Jing D, Buser D, Sculean A, Chandad F, Miron RJ (2016). Bone grafting material in combination with Osteogain for bone repair: a rat histomorphometric study. Clin Oral Investig, 20:589-595. 10.1007/s00784-015-1532-2 26174082

[159] Shreehari AK, Darekar HS, Borthakur R (2016). A comparative analysis of root surface biomodification with ethylene diamine tetra acetic acid and tetracycline hydrochloride: An in vitro scanning electron microscopic study. Med J Armed Forces India, 72:145-151. 27274611 10.1016/j.mjafi.2015.03.002 PMC4878859

[160] Choi JI, Seymour GJ (2010). Vaccines against periodontitis: a forward-looking review. J Periodontal Implant Sci, 40:153-163. 20827324 10.5051/jpis.2010.40.4.153 PMC2931303

[161] Timmerman A, Calache H, Parashos P (2017). A cross sectional and longitudinal study of endodontic and periapical status in an Australian population. Aust Dent J, 62:345-354. 28271515 10.1111/adj.12512

[162] Ahovuo-Saloranta A, Forss H, Hiiri A, Nordblad A, Makela M (2016). Pit and fissure sealants versus fluoride varnishes for preventing dental decay in the permanent teeth of children and adolescents. Cochrane Database Syst Rev, 2016:CD003067. 26780162 10.1002/14651858.CD003067.pub4 PMC7177291

[163] Zhang W, Chen X, Fan MW, Mulder J, Huysmans MC, Frencken JE (2014). Do light cured ART conventional high-viscosity glass-ionomer sealants perform better than resin-composite sealants: a 4-year randomized clinical trial. Dent Mater, 30:487-492. 24602520 10.1016/j.dental.2014.01.016

[164] Lokhande NA, Padmai AS, Rathore VP, Shingane S, Jayashankar DN, Sharma U (2014). Effectiveness of flowable resin composite in reducing microleakage - an in vitro study. J Int Oral Health, 6:111-114. PMC4109237 25083045

[165] Sadeghi M (2012). An in vitro microleakage study of class V cavities restored with a new self-adhesive flowable composite resin versus different flowable materials. Dent Res J (Isfahan), 9:460-465. 23162589 PMC3491335

[166] Banerji S, Mehta SB, Millar BJ (2010). Cracked tooth syndrome. Part 2: restorative options for the management of cracked tooth syndrome. Br Dent J, 208:503-514. 20543791 10.1038/sj.bdj.2010.496

[167] Gambeta E, Chichorro JG, Zamponi GW (2020). Trigeminal neuralgia: An overview from pathophysiology to pharmacological treatments. Mol Pain, 16:1744806920901890. 31908187 10.1177/1744806920901890 PMC6985973

[168] Xie E, Garzon-Muvdi T, Bender M, Doshi T, Carson B, Lim M, et al. (2019). Association Between Radiofrequency Rhizotomy Parameters and Duration of Pain Relief in Trigeminal Neuralgia Patients with Recurrent Pain. World Neurosurg, 129:e128-e133. 31102773 10.1016/j.wneu.2019.05.059

[169] Tavakol S, Jackanich A, Strickland BA, Marietta M, Ravina K, Yu C, et al. (2020). Effectiveness of Gamma Knife Radiosurgery in the Treatment of Refractory Trigeminal Neuralgia: A Case Series. Oper Neurosurg (Hagerstown), 18:571-576. 31620790 10.1093/ons/opz311

[170] Fischer LA, Demerath E, Bittner-Eddy P, Costalonga M (2019). Placental colonization with periodontal pathogens: the potential missing link. Am J Obstet Gynecol, 221:383-392 e383. 31051120 10.1016/j.ajog.2019.04.029 PMC6821581

[171] Chen CK, Wu YT, Chang YC (2017). Association between chronic periodontitis and the risk of Alzheimer's disease: a retrospective, population-based, matched-cohort study. Alzheimers Res Ther, 9:56. 28784164 10.1186/s13195-017-0282-6 PMC5547465

[172] Tada A, Senpuku H, Motozawa Y, Yoshihara A, Hanada N, Tanzawa H (2006). Association between commensal bacteria and opportunistic pathogens in the dental plaque of elderly individuals. Clin Microbiol Infect, 12:776-781. 16842573 10.1111/j.1469-0691.2006.01497.x

[173] Gonsalves WC, Wrightson AS, Henry RG (2008). Common oral conditions in older persons. Am Fam Physician, 78:845-852. 18841733

[174] Noble JM, Scarmeas N, Papapanou PN (2013). Poor oral health as a chronic, potentially modifiable dementia risk factor: review of the literature. Curr Neurol Neurosci Rep, 13:384. 23963608 10.1007/s11910-013-0384-x PMC6526728

[175] Maldonado A, Laugisch O, Burgin W, Sculean A, Eick S (2018). Clinical periodontal variables in patients with and without dementia-a systematic review and meta-analysis. Clin Oral Investig, 22:2463-2474. 10.1007/s00784-018-2523-x 29934798

[176] Koren O, Spor A, Felin J, Fak F, Stombaugh J, Tremaroli V, et al. (2011). Human oral, gut, and plaque microbiota in patients with atherosclerosis. Proc Natl Acad Sci U S A, 108 Suppl 1:4592-4598. 20937873 10.1073/pnas.1011383107 PMC3063583

[177] Shi T, Min M, Sun C, Zhang Y, Liang M, Sun Y (2018). Periodontal disease and susceptibility to breast cancer: A meta-analysis of observational studies. J Clin Periodontol, 45:1025-1033. 29974484 10.1111/jcpe.12982

[178] Przybylowska D, Mierzwinska-Nastalska E, Swoboda-Kopec E, Rubinsztajn R, Chazan R (2016). Potential respiratory pathogens colonisation of the denture plaque of patients with chronic obstructive pulmonary disease. Gerodontology, 33:322-327. 25393518 10.1111/ger.12156

[179] Fukugaiti MH, Ignacio A, Fernandes MR, Ribeiro Junior U, Nakano V, Avila-Campos MJ (2015). High occurrence of Fusobacterium nucleatum and Clostridium difficile in the intestinal microbiota of colorectal carcinoma patients. Braz J Microbiol, 46:1135-1140. 26691472 10.1590/S1517-838246420140665 PMC4704648

[180] Saeb ATM, Al-Rubeaan KA, Aldosary K, Udaya Raja GK, Mani B, Abouelhoda M, et al. (2019). Relative reduction of biological and phylogenetic diversity of the oral microbiota of diabetes and pre-diabetes patients. Microb Pathog, 128:215-229. 30625362 10.1016/j.micpath.2019.01.009

[181] Gao S, Li S, Ma Z, Liang S, Shan T, Zhang M, et al. (2016). Presence of Porphyromonas gingivalis in esophagus and its association with the clinicopathological characteristics and survival in patients with esophageal cancer. Infect Agent Cancer, 11:3. 26788120 10.1186/s13027-016-0049-x PMC4717526

[182] Hu J, Han S, Chen Y, Ji Z (2015). Variations of Tongue Coating Microbiota in Patients with Gastric Cancer. Biomed Res Int, 2015:173729. 26457297 10.1155/2015/173729 PMC4589578

[183] Li Y, Saxena D, Chen Z, Liu G, Abrams WR, Phelan JA, et al. (2014). HIV infection and microbial diversity in saliva. J Clin Microbiol, 52:1400-1411. 24523469 10.1128/JCM.02954-13 PMC3993673

[184] Ninomiya M, Hashimoto M, Yamanouchi K, Fukumura Y, Nagata T, Naruishi K (2020). Relationship of oral conditions to the incidence of infective endocarditis in periodontitis patients with valvular heart disease: a cross-sectional study. Clin Oral Investig, 24:833-840. 10.1007/s00784-019-02973-2 31197658

[185] Carinci F, Martinelli M, Contaldo M, Santoro R, Pezzetti F, Lauritano D, et al. (2018). Focus on periodontal disease and development of endocarditis. J Biol Regul Homeost Agents, 32:143-147. 29460534

[186] Carrizales-Sepulveda EF, Ordaz-Farias A, Vera-Pineda R, Flores-Ramirez R (2018). Periodontal Disease, Systemic Inflammation and the Risk of Cardiovascular Disease. Heart Lung Circ, 27:1327-1334. 29903685 10.1016/j.hlc.2018.05.102

[187] Pickard JM, Zeng MY, Caruso R, Nunez G (2017). Gut microbiota: Role in pathogen colonization, immune responses, and inflammatory disease. Immunol Rev, 279:70-89. 28856738 10.1111/imr.12567 PMC5657496

[188] Yang B, Petrick JL, Abnet CC, Graubard BI, Murphy G, Weinstein SJ, et al. (2017). Tooth loss and liver cancer incidence in a Finnish cohort. Cancer Causes Control, 28:899-904. 28534090 10.1007/s10552-017-0906-y PMC5639923

[189] Qin N, Yang F, Li A, Prifti E, Chen Y, Shao L, et al. (2014). Alterations of the human gut microbiome in liver cirrhosis. Nature, 513:59-64. 25079328 10.1038/nature13568

[190] Yan X, Yang M, Liu J, Gao R, Hu J, Li J, et al. (2015). Discovery and validation of potential bacterial biomarkers for lung cancer. Am J Cancer Res, 5:3111-3122. 26693063 PMC4656734

[191] Pessi T, Karhunen V, Karjalainen PP, Ylitalo A, Airaksinen JK, Niemi M, et al. (2013). Bacterial signatures in thrombus aspirates of patients with myocardial infarction. Circulation, 127:1219-1228, e1211-1216. 23418311 10.1161/CIRCULATIONAHA.112.001254

[192] Wu Y, Chi X, Zhang Q, Chen F, Deng X (2018). Characterization of the salivary microbiome in people with obesity. PeerJ, 6:e4458. 29576948 10.7717/peerj.4458 PMC5858547

[193] Ko CY, Liu QQ, Su HZ, Zhang HP, Fan JM, Yang JH, et al. (2019). Gut microbiota in obstructive sleep apnea-hypopnea syndrome: disease-related dysbiosis and metabolic comorbidities. Clin Sci (Lond), 133:905-917. 30957778 10.1042/CS20180891 PMC6465302

[194] Fan X, Alekseyenko AV, Wu J, Peters BA, Jacobs EJ, Gapstur SM, et al. (2018). Human oral microbiome and prospective risk for pancreatic cancer: a population-based nested case-control study. Gut, 67:120-127. 27742762 10.1136/gutjnl-2016-312580 PMC5607064

[195] Trikudanathan G, Philip A, Dasanu CA, Baker WL (2011). Association between Helicobacter pylori infection and pancreatic cancer. A cumulative meta-analysis. JOP, 12:26-31. 21206097

[196] Lindheim L, Bashir M, Munzker J, Trummer C, Zachhuber V, Pieber TR, et al. (2016). The Salivary Microbiome in Polycystic Ovary Syndrome (PCOS) and Its Association with Disease-Related Parameters: A Pilot Study. Front Microbiol, 7:1270. 27610099 10.3389/fmicb.2016.01270 PMC4996828

[197] Maresz KJ, Hellvard A, Sroka A, Adamowicz K, Bielecka E, Koziel J, et al. (2013). Porphyromonas gingivalis facilitates the development and progression of destructive arthritis through its unique bacterial peptidylarginine deiminase (PAD). PLoS Pathog, 9:e1003627. 24068934 10.1371/journal.ppat.1003627 PMC3771902

[198] Konig MF, Abusleme L, Reinholdt J, Palmer RJ, Teles RP, Sampson K, et al. (2016). Aggregatibacter actinomycetemcomitans-induced hypercitrullination links periodontal infection to autoimmunity in rheumatoid arthritis. Sci Transl Med, 8:369ra176. 10.1126/scitranslmed.aaj1921 PMC5384717 27974664

[199] de Smit MJ, Westra J, Brouwer E, Janssen KM, Vissink A, van Winkelhoff AJ (2015). Periodontitis and Rheumatoid Arthritis: What Do We Know? J Periodontol, 86:1013-1019. 25968957 10.1902/jop.2015.150088

[200] Mehrazarin S, Oh JE, Chung CL, Chen W, Kim RH, Shi S, et al. (2011). Impaired odontogenic differentiation of senescent dental mesenchymal stem cells is associated with loss of Bmi-1 expression. J Endod, 37:662-666. 21496667 10.1016/j.joen.2011.02.009 PMC3079884

[201] Macrin D, Alghadeer A, Zhao YT, Miklas JW, Hussein AM, Detraux D, et al. (2019). Metabolism as an early predictor of DPSCs aging. Sci Rep, 9:2195. 30778087 10.1038/s41598-018-37489-4 PMC6379364

[202] Cheng LB, Li KR, Yi N, Li XM, Wang F, Xue B, et al. (2017). miRNA-141 attenuates UV-induced oxidative stress via activating Keap1-Nrf2 signaling in human retinal pigment epithelium cells and retinal ganglion cells. Oncotarget, 8:13186-13194. 28061435 10.18632/oncotarget.14489 PMC5355087

[203] Chen L, Xin ZC, Li X, Tian L, Yuan YM, Liu G, et al. (2006). Cox7a2 mediates steroidogenesis in TM3 mouse Leydig cells. Asian J Androl, 8:589-594. 16752004 10.1111/j.1745-7262.2006.00178.x

[204] Li YG, Zhu W, Tao JP, Xin P, Liu MY, Li JB, et al. (2013). Resveratrol protects cardiomyocytes from oxidative stress through SIRT1 and mitochondrial biogenesis signaling pathways. Biochem Biophys Res Commun, 438:270-276. 23891692 10.1016/j.bbrc.2013.07.042

[205] Synowiec E, Szaflik J, Chmielewska M, Wozniak K, Sklodowska A, Waszczyk M, et al. (2012). An association between polymorphism of the heme oxygenase-1 and -2 genes and age-related macular degeneration. Mol Biol Rep, 39:2081-2087. 21647550 10.1007/s11033-011-0955-3 PMC3271228

[206] Siendones E, SantaCruz-Calvo S, Martin-Montalvo A, Cascajo MV, Ariza J, Lopez-Lluch G, et al. (2014). Membrane-bound CYB5R3 is a common effector of nutritional and oxidative stress response through FOXO3a and Nrf2. Antioxid Redox Signal, 21:1708-1725. 24450884 10.1089/ars.2013.5479 PMC4186635

[207] Lee YH, Kim GE, Cho HJ, Yu MK, Bhattarai G, Lee NH, et al. (2013). Aging of in vitro pulp illustrates change of inflammation and dentinogenesis. J Endod, 39:340-345. 23402504 10.1016/j.joen.2012.10.031

[208] Feng X, Feng G, Xing J, Shen B, Tan W, Huang D, et al. (2014). Repeated lipopolysaccharide stimulation promotes cellular senescence in human dental pulp stem cells (DPSCs). Cell Tissue Res, 356:369-380. 24676500 10.1007/s00441-014-1799-7

[209] Feng X, Xing J, Feng G, Huang D, Lu X, Liu S, et al. (2014). p16(INK4A) mediates age-related changes in mesenchymal stem cells derived from human dental pulp through the DNA damage and stress response. Mech Ageing Dev, 141-142:46-55. 25304494 10.1016/j.mad.2014.09.004

[210] Porrelli D, Gruppuso M, Vecchies F, Marsich E, Turco G (2021). Alginate bone scaffolds coated with a bioactive lactose modified chitosan for human dental pulp stem cells proliferation and differentiation. Carbohydr Polym, 273:118610. 34561009 10.1016/j.carbpol.2021.118610

[211] Liu H, Li W, Shi S, Habelitz S, Gao C, Denbesten P (2005). MEPE is downregulated as dental pulp stem cells differentiate. Arch Oral Biol, 50:923-928. 16183369 10.1016/j.archoralbio.2005.03.003

